# Ecological, morpho-agronomical, and bromatological assessment of sorghum ecotypes in Northern Morocco

**DOI:** 10.1038/s41598-023-41565-9

**Published:** 2023-09-20

**Authors:** S. Boukrouh, A. Noutfia, N. Moula, C. Avril, J. Louvieaux, J.L. Hornick, J.F. Cabaraux, M. Chentouf

**Affiliations:** 1 Department of Veterinary Management of Animal Resources, FARAH Center, Faculty of Veterinary Medicine, University of Liège, 4000 Liège, Belgium; 2 Regional Center of Agricultural Research of Tangier, National Institute of Agricultural Research, 10090 Rabat, Morocco; 3 Haute École Provinciale de Hainaut Condorcet, Agronomy Category, 7800 Ath, Belgium

**Keywords:** Plant sciences, Plant breeding, Plant ecology, Biodiversity

## Abstract

*Sorghum Bicolor* is a cereal used for grains as feed and food, mainly cultivated in dry areas. To study the possibilities of increasing its cultivation for feed purposes, ecological, morpho-agronomical, and bromatological characterization of some local ecotypes was conducted as the first steps toward selecting better cultivars. Indeed, twenty-one ecotypes were collected from farms in Northern Morocco in 2018. The edapho-climatic parameters of the collection sites were evaluated. The ecotypes were cultivated in 2019 in an experimental field with a randomized complete block design with three replicates. At the maturity stage, plants were evaluated for agro-morphological parameters, and grains and straw (leaves and stems) were harvested and analyzed. The results indicated significant variations between ecotypes for almost all parameters and an interesting grain yield of 3.5 T/ha with a 176% yield variation. The nutritive value of grains was interesting compared to straw, especially for mean protein contents (10.5% DM) and organic matter digestibility (81.4%). The calculated genetic parameters emphasized the possibility of selecting highly productive and nutritive cultivars. Multivariate analysis clustered the ecotypes into five groups based on agro-morphological, bromatological, and antioxidant activity parameters; the third group was characterized by high grain-yielding ecotypes, and the fifth one by high nutritive ecotypes. The E21 ecotype, belonging to this last group, was a promising selection candidate as it combines both. No significant correlation link between agro-morphological and bromatological traits of grains and geographical distances was discerned. Sorghum bicolor could thus be improved only according to the researched agro-morphological and bromatological traits.

## Introduction

Nowadays, coping with climate change is a major challenge, especially in dry areas where plant breeding faces several obstacles. Arid regions are characterized by limited resources, including water and nutrients^1^. The challenge of developing more resource-use-efficient cultivars is important for improving crop productivity and sustainability, especially since drought is expected to become more frequent and severe due to climate change. Thus, developing cultivars resilient to changing conditions is a priority for plant breeders to maintain crop productivity and food security.

*Sorghum bicolor* (L.) Moench, usually called sorghum, is the fifth leading cereal crop in the world after wheat, maize, rice, and barley^2^. It is a multi-purpose crop used as food (grains), feed (grains and fodder), and energy resource^3^. It is characterized by high fodder yield in a short time span, requires lower fertilizer inputs than corn, and has the particularity of wide adaptability to various agro-climatic conditions^4^.

Sorghum originated and was domesticated in Africa (about 5,000–8,000 years ago), where the highest diversity of cultivars was found^5^. Currently, it is cultivated on 28.42 million hectares of land, with production reaching 28.61 million tons per year^2^, thus, having an average yield of 1 T/ha.

Sorghum grains are known for their high content of minerals, vitamins, carbohydrates, other anti-nutritional secondary compounds such as phenolic compounds and tannins, and their antioxidant activity^6^. Other studies^7^ reported numerous genotypes with specific sizes (high, medium, or low), cycles (early or late), and aptitude (forage, grain, or dual-purpose), having a strong influence on the nutritional value of the fodder and grains. Djé et al*.*^7^ assessed the level of genetic diversity of Northern Moroccan sorghum ecotypes based on direct field sampling using allozyme and microsatellite markers and suggested that individual sorghum fields constitute valuable conservation units.

In Morocco, sorghum is used as a staple animal feed for cattle, sheep, and poultry for its grains, especially as a supplement in the summer season. It is also used as an energy source in the finishing phase of the fattening of calves and sheep^8^. It is conducted in rainfed conditions in marginalized areas as a secondary crop^8^. In 2021, the Moroccan farmers produced quantities reached 5,000 tons in an area of 4,600 ha, with a mean yield of 1.1 T/ha^2^, ranging from 200 kg to 2 T/ha^8^. Low yields are due to bad weather conditions, traditional agricultural techniques based on old methods, and parceled-out lands^9^. The phenotypic selection exerted by farmers since the introduction of sorghum in Morocco has engendered locally adapted ecotypes^9^. The ease of farming techniques encouraged its cultivation^8^.

Due to climate change with more prolonged drought periods, the decrease in water supply, and the increasing world population, this drought-resistant crop, like other available local feed alternatives^10^, could quickly become crucial and see its global use increase. Previous studies emphasized the agro-morphological and genetical diversity of some Moroccan ecotypes^7,11,12^. However, no studies assessed the nutritive value as feed on these ecotypes. This study collected twenty-one sorghum local germplasm from the traditional production area in the north of Morocco. It aimed thus to link the ecological and agro-morphological variability to the nutritive value (grain and straw) of twenty-one Moroccan sorghum ecotypes. This characterization could allow the preservation of local germplasms, help to choose the more productive and interesting ecotypes for feed, and enrich the biodiversity of the Moroccan sorghum gene bank.

## Materials and methods

All experiment and analysis methods were performed following relevant regulations and guidelines.

### Plant material, study site, and experimental set‑up

**Figure 1:**
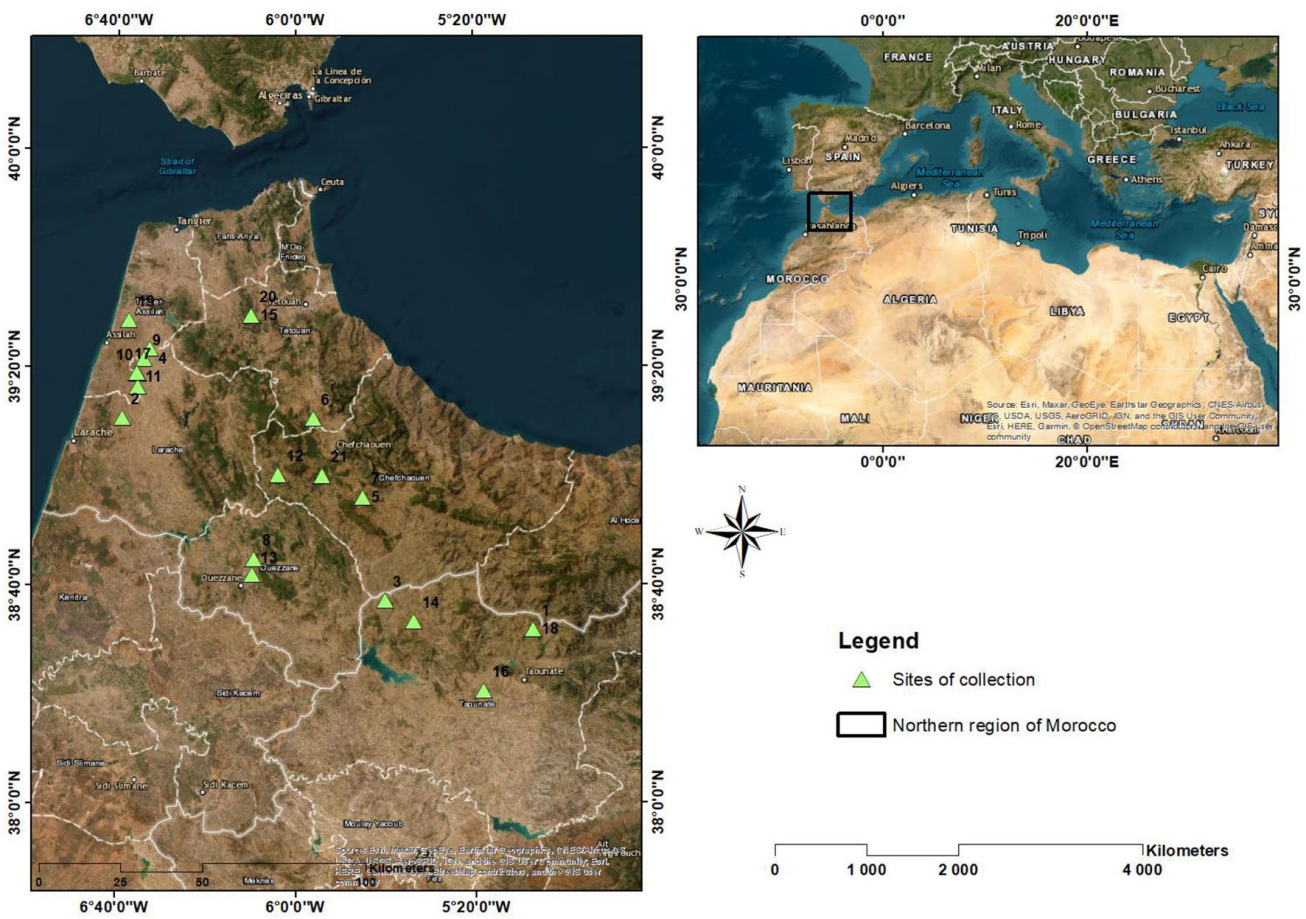
Collection sites of the 21 *Sorghum bicolor* ecotypes (this map was generated using ArcGIS Desktop version 10.4.1—Esri, Redlands, CA, USA, https://en.freedownloadmanager.org/Windows-PC/Portal-for-ArcGIS.html).

In 2018, seeds of 21 Sorghum ecotypes were collected using a simple random sampling from 21 farms in Northern Morocco (Fig. 1, Supplementary Table 1, Supplementary Figure). Prospection missions were conducted just after the end of the sorghum harvest season. In each farm, 3 kg of grains were collected from the grain stock.

The seeds were sown in 2019 in the El Menzla experimental field (35°31′53′′ N; 5°42′36′′ W; 128.5 m), a related research station to INRA of Tangier, Morocco.

### Ecological characterization

At the collection and experimental sites, five soil samples were collected from 0 to 20 cm and 20–40 cm horizons for physico-chemical analysis (Table 1, Supplementary Table 2﻿). Soil samples were oven dried at 60 °C until constant weight to determine soil humidity (%), then grounded and passed through a 2 mm sieve. The pH (water and KCl) was read with a standard calibrated pH meter in 2:1 distilled water solution and dry soil ratio^13^. The nitrogen was determined by mineralization and distillation using the Kjeldahl method^14^. The electrical conductivity was measured in the collected soil extract from the saturated soil paste by conductivity meter^15^. Exchangeable potassium was analyzed in 1 N ammonium acetate extract using a flame photometer^16^. Total limestone (CaCO_3_) was measured using HCl^17^. Carbon was measured through dichromate oxidation and converted to organic matter (OM) by multiplying by a factor of 1.72^18^, and available phosphorus was determined by the colorimetric method^19^. Soil texture was determined by using the standard Pipette method and wet sieving^20^.

**Table 1:** Climatic and physico-chemical characteristics of the soils of the 21 collection farms and the experimental cultivation site in Northern Morocco.

	Collection farms								Experimental site 2019	
	Mean		Min	Max	Standard deviation	1st quartile	Median	3rd quartile		
Altitude (m)	333.7		26.0	943.0	267.6	69.0	304.0	540.0	129.0	
Bioclimatic parameters										
Average annual temperature (BIO1, °C)	17.7		16.4	18.9	0.8	17.1	18.0	18.3	18.1	
Average diurnal variation (BIO2, °C)	10.7		8.6	11.9	1.2	9.7	11.4	11.6	22.9	
Isothermality (BIO3 = BIO2/BIO7 × 100, %)	40.3		39.2	41.0	0.5	40.0	40.3	40.7	57.3	
Temperature seasonality (BIO4)	545.8		443.5	618.1	63.8	486.7	571.9	600.9	542.1	
Maximum temperature of the warmest month (BIO5, °C)	32.2		29.3	35.1	1.8	30.6	32.7	33.8	40.7	
Minimum temperature of the coldest month (BIO6, C°)	5.7		3.7	8.2	1.4	4.3	5.7	6.8	0.7	
Temperature annual range (BIO7 = BIO5-BIO6, °C)	26.5		21.1	29.6	3.0	24.0	28.2	29.0	40.0	
Average temperature of the wettest quarter (BIO8, °C)	11.4		9.9	13.2	1.2	10.0	11.6	12.5	16.2	
Average temperature of the driest quarter (BIO9, °C)	24.5		23.2	26.6	0.9	24.0	24.2	25.0	21.8	
Average temperature of the warmest quarter (BIO10, °C)	24.7		23.4	26.6	0.7	24.3	24.6	25.0	24.4	
Average temperature of the coldest quarter (BIO11, °C)	11.3		9.4	13.2	1.3	10.0	11.6	12.4	11.8	
Annual precipitation (BIO12, mm)	784.0		627.0	940.0	94.0	742.0	774.0	864.0	563.4	
Precipitation of the wettest month (BIO13, mm)	140.4		97.0	169.0	20.5	134.0	142.0	153.0	152.0	
Precipitation of the driest month (BIO14, mm)	0.4		0.0	1.0	0.5	0.0	0.0	1.0	0.0	
Precipitation seasonality (BIO15, mm)	80.4		68.0	88.3	6.4	77.4	82.6	85.4	117.8	
Precipitation of the wettest quarter (BIO16, mm)	389.8		267.0	479.0	60.6	377.0	398.0	421.0	129.8	
Precipitation of the driest quarter (BIO17, mm)	12.1		7.0	18.0	3.4	9.0	13.0	15.0	0.1	
Precipitation of the warmest quarter (BIO18, mm)	16.6		13.0	20.0	1.9	16.0	17.0	17.0	1.3	
Precipitation of the coldest quarter (BIO19, mm)	389.4		261.0	479.0	61.1	377.0	398.0	421.0	44.9	
Edaphic parameters	0–20 cm	20–40 cm							0–20 cm	20–40 cm
Humidity (%)	5.9	5.8	3.6	9.5	1.6	5.0	5.9	6.6	6.2	4.2
pH water	7.9	7.9	4.5	8.7	1.1	7.9	8.4	8.5	8.2	8.6
pH KCl	7.5	7.5	4.4	8.5	1.1	7.4	7.6	8.3	7.1	7.7
Electrical conductivity (mS/m)	23.2	22.3	1.0	76.3	28.2	2.2	7.3	50.2	33.4	28.6
Organic matter (%)	3.1	2.9	0.7	5.2	1.3	2.0	2.8	4.0	2.4	2.0
Total limestone (CaCO_3_, %)	2.2	2.7	0.8	7.6	1.9	1.1	2.0	2.4	7.4	7.4
Exchangeable potassium (K, ppm)	91.1	90.3	4.1	391.8	103.4	13.0	24.8	165.2	740.6	670
Available phosphorus (P_,_ ppm)	81.1	65.6	32.0	128.5	37.7	35.0	77.6	107.0	42.6	36.8
Carbon to nitrogen ratio (C/N)	10.8	10.4	4.6	16.9	3.7	7.3	10.3	13.7	6.6	6.2
Nitrogen (N, %)	0.2	0.2	0.1	0.4	0.1	0.2	0.2	0.2	0.2	0.2
Clay (%)	30.7	31.0	0.0	82.5	28.2	0.0	27.9	56.2	46.7	53.3
Coarse sand (%)	4.4	4.5	0.2	19.2	6.2	0.4	0.7	4.8	1.9	1.3
Fine sand (%)	5.3	5.4	0.2	29.0	9.4	0.5	1.0	3.6	1.1	1.1
Coarse silt (%)	47.2	47.7	17.2	80.2	19.2	36.5	39.5	62.1	38.4	31.6
Fine silt (%)	12.1	11.1	0.0	24.5	7.3	6.7	10.0	20.0	11.9	8.1

Nineteen bioclimatic data (BIO 1–19) for 1950–2000 were estimated from major climate databases for each farm collection site (https://www.worldclim.org). The data were extracted from satellite images via ArcGIS Desktop version 9.3 (Esri, Redlands, CA, USA) and were interpolated with a resolution of approximately 1 km^21^ (Table 1). The climatic data (minimum, maximum, and mean temperatures (°C), rainfall (mm) of each month, and total rainfall (mm)) of the 2019 agronomical year at the experimental site were collected from a climatic station 10 km nearer. The 2019 bioclimatic variables (BIO 1–19) were determined based on these climatic data.

### Experimental design

The experiment was conducted under a randomized block design with three replicates. Ecotypes were sown on 10^th^ April 2019, with a density of 10 plants/m^2^ with a spacing of 50 cm between lines and 20 cm between plants in a 3 × 2 m^2^ plot. One meter was the distance between plots and between blocks. The trial was conducted under rainfed conditions on a fallow plot. As soil analysis indicated low phosphorus concentration compared to potassium and nitrogen, NPK (10-30-10) fertilizer was applied at the rate of 100 kg/ha on the day of sowing. No watering was needed at sowing because rainfall occurred immediately after.

### Agro-morphological characterization

#### Phenological assessment

Every three days, the phenological parameters were observed during the growing season. Days to flowering (DayF) and days to maturity (DayM) were recorded as the days from planting until 50% of the plants reached the flowering stage and the physiological maturity, respectively^1^. The grain filling period (GFP) was measured as the difference between these two parameters.

#### Morphological assessment

Fourteen quantitative and seventeen qualitative traits were recorded at the ecotype maturity stage. These traits were described based on the recommendations of the International Board for Plant Genetic Resources and the International Crops Research Institute for the Semi-Arid Tropics^22^, from five randomly individual plants per ecotype in each plot. The plant height (PH) was recorded as the height of the plant from the ground to the tip of the panicle. The peduncle length (PedL) was measured as the average exertion of the panicle from the flag leaf’s blade to the base of the lowest panicle branch. The leaves number (LN) and internodes number (IntN) were counted on each plant. The length and width of the leaf (LL and LWi, respectively) and the stem diameter (SD) were measured at the third internode. The panicle length (PanL) was recorded as the average length of the panicle from the lower panicle branch to the tip of the panicle, and the panicle width (PanWi) was measured as the average width of the panicle at its widest section. The panicle weight (PanW) was recorded as the weight of an unthreshed panicle. The panicles were threshed to evaluate the number of grains per plant (GPL). Ultimately, the plants were separated into their different parts, dried in an oven at 102 °C until constant weight, and were transformed into percentages of panicle (pPan), leaves (pLeav), and stems (pStem). The 18 qualitative variables were assessed for sorghum. Leaf rolling (non-rolled to fully rolled), color (dark and light green), and orientation (erect or drooping) were visually rated on the leaves of five identified plants at the flowering stage. Before harvesting, stay green (very slightly senescent, slightly senescent, intermediate (about half of the leaves are dead), mostly senescent, completely senescent leaves and dead stalk), and lodging susceptibility (low, intermediate, and high) were observed on five whole plants. Then after harvesting the panicles, the other parameters were evaluated using IBPGR and ICRISTAT descriptors^22^. Their compactness was classified into (very lax panicle, very loose erect primary branches, very loose drooping primary branches, loose erect primary branches, semi-loose erect primary branches, semi-loose drooping primary branches, semi-compact elliptic, compact elliptic, compact oval), and the exertion of the peduncle was classified to (slightly exserted, exserted, well exserted, peduncle semi-recurved, peduncle recurved). These two parameters were classified by assigning them to different shapes shown by the descriptors. The frequency of grain color (white, yellow, red, black, and brown), grain luster (present or absent), grain shape (narrow elliptic, elliptic, circular), grain size (small (< 5mm), middle (5–10 mm), and big (> 5 mm), aristation (present or absent), endosperm texture (mostly corneous, intermediate, mostly starchy, completely starchy), grain covering (25% of grain covered, 50% of grain covered, 75% of grain covered, full grain covered) and glume color (white, beige, purple, black, red), glume hairiness (present or absent) and shattering (low, intermediate or high) were evaluated on ten randomly selected grains per plant by visually comparing them to the morphological descriptors present in the IBPGR and ICRISAT options^22^.

#### Agronomical assessment

All the plants in a plot (n = 60 plants per plot) served to determine the grain yield (GRY) as the dry weight of the grains. Thousand seed weight (TSW) was recorded as the weight of one thousand seeds sampled thrice from bulked seeds in each plot. The straw was measured as the weight of the above-ground plant parts. The harvest index (HI) was calculated as the ratio of GRY to straw.

### Bromatological analysis

#### Chemical composition

At the maturity stage, the plots were harvested (n = 55 plants per plot) and separated into grains, leaves, and stems. The separated material was dried at 50 °C for 48h, ground, and passed through a 1 mm sieve for bromatological analysis. The samples were evaluated according to AOAC^23^ to determine the contents of dry matter (DM) by drying 5 g of the sample at 102 °C until constant weight (method 934.01), ash by incinerating 5 g of the sample at 550 °C for 12h (method 942.05), ether extract (EE) by extraction with diethyl ether in Soxhlet apparatus (method 963.15). The crude protein (CP) content was determined by multiplying the nitrogen content by 6.25, obtained after mineralization with H_2_SO_4_ and distillation with NaOH using the Kjeldahl method (method 977.02). Fiber content (crude fiber (CF), neutral detergent fiber (NDF), acid detergent fiber (ADF), and acid detergent lignin (ADL)) were analyzed using an ANKOM® 200 Fiber Analyzer (ANKOM Technology, Macedon, NY, USA), following the method of AOAC^24^ and Van Soest et al*.*^25^. The nitrogen-free extract (NFE) was estimated using the following “Eq. (1)”:

1 $${\text{NFE }}\left( {\% {\text{ DM}}} \right) \, = { 1}00 \, - \, \left( {{\text{EE }} + {\text{ CP }} + {\text{ CF }} + {\text{ Ash}}} \right).$$

Total phenols (TP) and total tannins (TT) were quantified in a methanol extract solution using the Folin–Ciocalteu method^26^. Total phenolic substances were determined by combining 40 µL of the sorghum methanol extract, 60 µL of distilled water, 50 µL of Folin–Ciocalteu reagent, and 225 µL of sodium carbonate (20%). The mixture was vortexed and kept in the dark for 40 min at room temperature. The tube was centrifuged, and an aliquot of 250 µL of the supernatant was transferred to a microplate well. The absorbance was read at 725 nm. Non-tannic phenols were determined in another centrifuge tube: 750 µL of the extract was mixed with 100 mg of polyvinylpolypyrrolidone (PVPP) and 1 mL of demineralized water. The tube was vortexed, kept at 4 °C for 15 min, centrifuged at 4 °C at 3,500 rpm for 10 min, and then read at 725 nm. Tannic acid at different concentrations (5–16 µg/mL) was used to construct the calibration curve. Total tannins were calculated as the difference between total and non-tannic phenols after precipitation with PVPP. The condensed tannins (CT) were analyzed by Porter et al*.* method^27^. Briefly, 3 mL of Butanol-HCl reagent were mixed in a glass tube with 0.1 mL of ferric reagent and 0.5 mL of the extract. The tubes were vortexed and put in a water bath at 97 °C for one hour. After cooling, the absorbance was read at 550 nm.

#### Digestibility

The in vitro enzymatic dry and organic matter digestibilities (IVEDMD and IVEOMD) were determined by the enzymatic method in a two-step method^28^. The first step concerned the incubation of 0.5 g of dry sample with 20 mL of a 2% pepsin solution diluted in 0.1 N hydrochloric acid. After 24 h of incubation at 40 °C, the sample was solubilized in 50 mL of a buffer solution containing 1 g/L cellulase and again incubated at 40 °C for 24 h. After incubation, the sample was rinsed with hot distilled water and then placed in an oven at 60 °C until constant weight. It was weighed to determine the IVEDMD. The sample was incinerated in the muffle furnace at 550 °C for 12 h to determine IVEOMD. The in vitro true digestibility (IVTD) was determined by incubation of feed samples in filter bags in a Daisy II incubator® (ANKOM Technology, Fairport, NY, USA)^29^. All the study procedures were approved by the Regional Center of Agricultural Research of Tangier (permit number: 01/CRRAT/2017). Rumen fluid was collected from five animals at a communal slaughterhouse. The animals were fed a conventional diet based on oat hay, barley, and fava bean grains, as distributed by regional farmers. The animals were slaughtered almost 12 h after feeding, and rumen fluid was immediately collected and sieved using a double cheese filter. Then, it was kept in a thermos at 39 °C to maintain the viability of rumen microflora. After arriving at the laboratory, it was added to artificial saliva in a 1:5 ratio containing samples heat-sealed in ANKOM F57 filter bags and incubated at 39.5 °C for 48h. The IVTD was estimated by quantifying residual DM compared to incubated initial quantities. The metabolizable energy (ME) was calculated according to AOAC^24^ using “Eq. (2)”:

2 $${\text{ME }}\left( {{\text{MJ}}/{\text{kg DM}}} \right) \, = \, 0.{17 } \times {\text{ IVEDMD }}{-}{ 2,}$$

where IVEDMD is the in vitro enzymatic dry matter digestibility in percentage. The in vitro enzymatic crude protein digestibility (IVECPD) was determined according to the procedure described by Aufrère and Cartailler^30^. Briefly, 1.0 g of ground sample was added to 50 mL of enzyme solution (0.1 g protease per 1L of borate–phosphate buffer; pH 6.8). Then, the tubes were sealed and incubated at 40 °C for 24 h under permanent stirring. Subsequently, samples were filtered, and residual N content was analyzed. The IVECPD was calculated according to the following “(Eq. 3)”:

3 $${\text{IVECPD}}(\% ) = \frac{{{\text{(N}}_{{{\text{sample}}}} - {\text{N}}_{{{\text{residue}}}} )}}{{{\text{N}}_{{{\text{sample}}}} }} \times 100,$$

where N_sample_ represents the nitrogen content of the sample, and N_residue_ represents the nitrogen remaining after digestion.

### Antioxidant activity

The grounded and sieved sorghum grains (2 g) were mixed with 20 mL of 80% methanol in a shaker at room temperature for 12 h. The resulting supernatants were filtered using Whatman filter paper and centrifuged at 6000 rpm for 10 min. Finally, the filtrate was evaporated at 30 °C. The dried extract was weighed and redissolved in methanol to a concentration of 1 mg/mL, then stored at 20 °C until analysis. The 2,2-diphenyl-1-picrylhydrazyl (DPPH) radical scavenging activity was determined according to the procedure reported by Tepe et al*.*^31^. For this purpose, 100 μL of various concentrations of the extract were added to 300 μL of a 100 mM methanol solution of DPPH. The absorbance was read after a 30 min incubation period against a blank at 517 nm at room temperature. Inhibition of free radical DPPH in percent (%) was calculated as follows “(Eq. 4)”:

4 $${\text{DPPH scavenging activity }}\left( \% \right) \, = \, \left[ {\left( {{\text{A}}_{{{\text{control}}}} - {\text{ A}}_{{{\text{sample}}}} } \right) \, /{\text{A}}_{{{\text{control}}}} } \right] \, \times { 1}00,$$

where A_control_ is the absorbance of DPPH in the absence of a sample and A_sample_ is the absorbance of DPPH in the presence of a sample. Extract concentration providing 50% inhibition (IC50) was calculated from the graph of inhibition percentage against extract concentration. The Ferric-reducing ability of plasma (FRAP) of grain extract was determined according to the method of Benzie and Strain^32^. Briefly, FRAP solution was prepared by mixing acetate buffer (300 mM, pH 3.6), TPTZ solution (10 mM in 40 mM HCl), and FeCl_3_.6H_2_O solution (20 mM) in a 10:1:1 ratio and was incubated at 30 °C for 30 min. Then, 40 µL of the extract solution was mixed with 360 µL of the freshly prepared FRAP solution. The mixture was shaken and incubated for 4 min at 37 °C in a water bath, and the absorbance was read at 593 nm.

### Data analysis

Analysis of variance was carried out to test the ecotype effect. The variance components were estimated using a general linear model (GLM), using SAS 9.4 version (SAS Inst. Inc., Cary, NC, USA). Phenotypic coefficients of variation (PCV), genotypic coefficients of variation (GCV), broad-sense heritability (H^2^), genetic advance, genetic advance as a percentage of the mean (GAM), and genotypic and phenotypic correlation matrix were estimated following the formula given by Shariatipour et al*.*^33^. All genetic parameters were estimated using the following Eqs. “(5, 6, 7, 8, 9, 10, 11)”:

5 $${\upsigma }^{2}\mathrm{g}=\frac{(MSg-MSe)}{r},$$

6 $${\upsigma }^{2}\mathrm{p}=\frac{MSg}{r},$$

7 $$\mathrm{GCV } (\%)=\frac{\sqrt{\upsigma ^{2}\mathrm{g}}}{\bar{\mathrm{x}}}\times 100,$$

8 $$\mathrm{PCV }(\%)=\frac{\sqrt{\upsigma ^{2}\mathrm{p}}}{\bar{\mathrm{x}}}\times 100,$$

9 $${\mathrm{H}}^{2} (\%)=\frac{\upsigma ^{2}\mathrm{g}}{\upsigma ^{2}\mathrm{p}}\times 100,$$

10 $$\mathrm{GA}= \mathrm{H}^{2} \times \mathrm{k }\times \sqrt{\upsigma ^{2}\mathrm{p}},$$

11 $$\mathrm{GAM }(\%) =\frac{\mathrm{GA}}{\bar{\mathrm{x}}}\times 100,$$

where ${\upsigma }^{2}\mathrm{g}$ is the genotypic variance, MSg is the mean square of genotypes, MSe is the mean square of error, r is the number of replications, ${\upsigma }^{2}\mathrm{p}$ is the phenotypic variance, x̅ is the general mean of the trait, and k is the selection differential, which is equal to 2.06 at 5% intensity of selection. To summarize and visualize the relationship between agro-morphological and bromatological parameters, a correlation matrix was generated using the “corrplot” package of R software, version 4.2.1. Principal component analysis (PCA) was performed using the “FactoMineR” and “Factoextra” packages. A heatmap was created using the “Pheatmap” package, with Euclidean distance as the similarity measure and hierarchical clustering with complete linkage. The Mantel test was used to assess the correlation between the phenotypic distance of the ecotypes with the geographic and environmental one. The Tidyverse”, “Vegan”, and “Geosphere” packages were used.

## Results and discussion

### Ecological characterization

The soil results concerning the collection and experimental sites are shown in Table 1. The collection sites were located on different altitudes varying from 26 to 943 m. The results showed that sorghum ecotypes could grow on various ranges of soil pH (4.5–8.7 for pH water and 4.4–8.5 for pH KCl), electrical conductivity (1.0–76.3 mS/m), organic matter (0.7–5.2%) and total limestone (0.8–7.6%). One author reported that Sorghum plants cannot grow well in acidic soils^34^. In the present study, the observation of sorghum cultivation on acidic soils in some farms could nuance these results. Sorghum was also found on a variable range of soil parameters related to nitrogen (0.1–0.4%), carbon to nitrogen ratio (4.6–16.9), exchangeable potassium (4.1–391.8 ppm), and available phosphorus (32.0–128.5 ppm). These variations are obviously due to the differences in farm fertilization strategies. Compared to the farms, the nitrogen was similar, exchangeable K higher (705.3 vs. 90.7 ppm), and available phosphorus lower (39.7 vs 73.5 ppm) in the experimental station soil. Phosphorus being the second most growth-limiting macronutrient after nitrogen, fertilizer was applied to correct the difference on the sowing day. The textural composition also varied for clay (0.0–82.5%), coarse sand (0.2–19.2%), fine sand (0.2–29.0%), coarse silt (17.2–80.2%), and fine silt (0.0–24.5%). Similar variability was observed between different sites where Ethiopian sorghum landraces were collected^35^. The 2019 annual precipitations were lower than the 1950–2000 mean values of the collection sites (563.4 vs. 784 mm), which could expose ecotypes to drought stress conditions at the experimental site.

### Qualitative parameters

The frequencies of the different qualitative traits of the tested plants are reported in Table 2. The knowledge of phenotypic diversity among sorghum is an essential tool for their efficient utilization in plant breeding models and effective conservation. Leaf rolling, a mechanism to reduce water evaporation when water stress is present^36^, was absent in 87.3% of the plants. It could be explained by optimal climatic conditions or drought resistance due to osmotic adjustment^37^. Peduncle exertion is also an indication of water stress occurrence^38^. In the present study, only one-third of the plants had recurved peduncles. The 2019 precipitations during the growing season were 28% lower than the long-term average, corroborating the plant’s drought resistance hypothesis. Stay green is another characteristic of drought resistance, measuring the ability of the plant to retain greenness during grain ripening under water-limited conditions^37^. In the present study, less than 2% of the plants were completely senescent, while almost 70% were very slightly senescent. The north of Morocco is a region where high wind speeds routinely occur^39^. The lodging resistance is an important parameter that should be targeted, especially in lowlands where machines harvest. Fortunately, more than 80% of plants manifested low lodging susceptibility. For leaves color, almost all of the plants (95.2%) had dark green leaves, which testify to the probable higher photosynthesis activity. It could also be reinforced by the erect orientation of the leaves (74.0%).

**Table 2:** Frequencies of the different qualitative parameters considered to describe the 21 cultivated Moroccan Sorghum ecotypes.

Parameters and codes	Variables and scores	Frequency (%)
Leaf rolling	Non rolled leaves	87.3
	25% of rolled leaves	7.9
	50% of rolled leaves	4.8
Stay green	Very slightly senescent	68.3
	Slightly senescent	15.6
	Intermediate (about half of the leaves are dead)	10.5
	Mostly senescent	4.1
	Completely senescent leaves and dead stalk	1.6
Leaf color	Dark green	95.2
	Light green	4.8
Leaf orientation	Erect	74.0
	Drooping	26.0
Lodging susceptibility	Low	82.2
	Intermediate	14.0
	High	3.8
Midrib color	White	85.0
	Light green	15.0
Panicle compactness	Very lax panicle	4.4
	Very loose erect primary branches	2.2
	Very loose drooping primary branches	0.6
	Loose erect primary branches	3.5
	Semi-loose erect primary branches	27.6
	Semi-loose drooping primary branches	1.0
	Semi-compact elliptic	23.2
	Compact elliptic	33.3
	Compact oval	4.1
Exertion peduncle	Slightly exserted	30.8
	Exserted	4.8
	Well exserted	29.8
	Peduncle semi-recurved	4.4
	Peduncle recurved	30.2
Grain color	White	45.6
	Yellow	25.8
	Red	20.7
	Black	5.3
	brown	2.6
Grain luster	Present	7.3
	Absent	92.7
Grain shape	Narrow elliptic	10.1
	Elliptic	45.7
	Circular	44.3
Grain size	Small (< 5mm)	20.0
	Middle (5–10 mm)	80.0
	Big (> 5 mm)	0.0
Aristation	Present	12.7
	Absent	87.3
Endosperm texture	Mostly corneous	4.8
	Intermediate	4.1
	Mostly starchy	66.7
	Completely starchy	24.4
Grain covering	25% of grain covered	13.0
	50% of grain covered	26.7
	75% of grain covered	52.7
	Full grain covered	7.7
Glume color	White	22.2
	Beige	35.0
	Purple	14.7
	Black	18.5
	Red	9.6
Glume hairiness	Present	47.3
	Absent	52.7
Shattering	Low	10.1
	Intermediate	14.5
	High	75.4

In the literature, midrib color can be white, light and dark green, brown, or yellow^40^. More than 80% of the leaves had white midrib. Green midrib color was reported to indicate juicy stems, while white midrib color indicates pithy stems, i.e., less palatable and less digestible^41^. Almost two-thirds of the plants had semi-compact and compact elliptic branches for panicle compactness. It could be due to the selection by the farmers for high-yielding cultivars. Grain colors were also variable, with almost half of the plants having a white color. Grain luster was absent in more than 90% of the plants, and 80% of the plants had middle-size grains. Half of the plants had circular, and the other half had elliptic grains shape. For grain covering, only less than 8% of grains were fully covered; some had glumes longer than grains. The fact that the major part of grains were not fully covered seems to be a selected feature that facilitates drying in order to minimize grain mold^42^. It could also facilitate shattering, as observed with about 75% of the panicles. Glumes color also varied, and almost 57% were pale. However, darker glumes are reportedly more resistant to grain mold incidence^43^. Half of the glumes were hairy, while more than 90% had an absence of aristation. Almost 70% of the grains had mostly starchy endosperm.

### Quantitative parameters

#### Agro-morphological characterization

For agro-morphological parameters, the variation between the ecotypes was statistically significant (p < 0.05) for all the traits, indicating considerable phenotypic variability between the ecotypes (Table 3, Supplementary Table 3﻿). The selection of performant ecotypes is usually based on phenotypic characterization, and the success would naturally depend upon the relationship between the phenotype and the genotype. To explain the variability, Chithra et al*.*^44^ used the estimated genotypic and phenotypic coefficients of variation (GCV and PCV) and the degree of heritability. Heritability (H^2^) estimates remain extremely useful in studying the inheritance of quantitative traits following selection and deciding suitable breeding procedures for improving a crop plant. The selection could be more effective for a specific trait improvement by using both H^2^ and genetic advance than the unique use of H^2^. So, GCV and PCV are categorized as low (0–10%), moderate (10–20%), and high (> 20%)^44^. Most of the parameters in the present study, including plant height, percentage of leaves, peduncle length, panicle length, width and weight, grain filling period, grains per plant and yield, harvest index, and thousand seed weight, had high GCV and PCV which indicates that a selection could be applied based on those traits to isolate more promising cultivars. The number of leaves and internodes, leaf length, stem diameter, and days to flowering exhibited intermediate GCV and PCV, which suggested that a vigorous selection could improve these traits. The days to maturity had low GCV and PCV, indicating less variability for this character and low options for breeders to diverse varieties for this trait. The heritability (H^2^) is categorized as low (0–30%), moderate (30–60%), and high (60% and above)^44^. All the parameters were highly heritable except for leaf width and stem diameter, which had moderate heritability. Therefore, the response to direct selection could successfully improve all these traits. The observed variation in a population is due to both genetics and environmental factors, whereas genetic variability is the only heritable part from generation to generation. Thus, heritability alone does not provide an idea about the expected gain in the next generation, but it must be considered in conjunction with the genetic advance. According to Chitra et al*.*^44^, genetic advance as a percent of the mean (GAM) is classified as low (< 10%), moderate (10–20%), and high (> 20%). Among the characters under study, except for the number of leaves and days to maturity that showed moderate GAM and leaf width that showed low GAM, all the other parameters exhibited high GAM coupled with high heritability. It could be used as a powerful tool in the selection process as such characters are controlled by the additive genes and are less influenced by the environment. Hence, the direct selection of such traits could also be effective in improving the yield. Close results were reported with Ethiopian ecotypes^43^. On the other hand, low to moderate heritability and genetic advance values could hinder the selection due to high environmental effects over the genetic effects^44^. So, only an effective selection can be obtained with the traits having higher GCV, PCV, H^2^, and GAM, meaning that the additive genetic effects are sufficiently robust to the environmental effect. The improvement of the traits with low heritability and genetic advance can also be boosted over heterosis breeding.

**Table 3:** Descriptive statistics and genetic parameters of 20 quantitative agro-morphological traits in 21 sorghum studied ecotypes.

Trait	Max	Min	Mean	SEM	GCV	PCV	H^2^	GA	GAM
Plant height (cm)	168.9	43.0	108.5***	1.8	22.6	23.7	91.0	48.1	44.4
Internodes number	11.0	5.8	7.6***	0.1	12.6	14.2	78.1	1.7	22.9
Leaves number	10.4	5.2	6.7***	0.1	11.2	13.0	73.7	1.3	19.8
Leaf length (cm)	43.8	17.5	31.7***	0.4	13.7	15.1	82.0	8.1	25.6
Leaf width (cm)	6.8	2.5	3.4***	0.1	6.2	12.1	26.4	0.2	6.6
Stem diameter (cm)	1.6	0.6	0.8*	0.0	14.1	19.1	54.1	0.2	21.3
Percentage of leaves (%)	18.2	3.1	7.6***	0.2	37.7	40.8	85.5	8.3	71.8
Percentage of stems (%)	24.0	2.4	41.4***	0.4	17.6	20.5	73.8	12.9	31.1
Percentage of panicles (%)	68.6	16.5	47.1***	0.7	17.8	20.5	75.6	15.0	31.9
Peduncle length (cm)	81.7	15.6	36.0***	1.0	38.2	39.5	93.3	27.3	76.0
Panicle length (cm)	30.9	8.7	17.6***	0.3	26.3	27.0	94.6	9.3	52.6
Panicle width (cm)	9.7	3.0	6.6***	0.1	23.7	25.6	85.7	3.0	45.3
Panicle weight (g)	112.1	3.6	39.5***	1.5	40.8	46.6	76.6	29.0	73.5
Days to flowering (days)	117.0	65.0	81.6***	1.6	15.3	15.4	98.0	25.4	31.1
Days to maturity (days)	142.0	94.0	113.0***	1.3	9.3	9.5	95.9	21.2	18.8
Grain filling period (days)	55.0	20.0	31.4***	1.1	27.8	28.1	98.1	17.8	56.7
Grains per plant	3,485.6	182.6	1,472.9***	93.4	42.9	44.8	91.9	1,248.8	84.8
Grain yield (T/ha)	7.7	0.5	3.5***	0.2	48.7	53.2	84.0	3,260.2	92.0
Harvest index (%)	75.0	13.6	51.8***	1.8	27.6	28.6	93.6	28.6	55.1
Thousand seed weight (g)	34.4	12.7	22.5***	0.8	26.2	26.8	95.9	11.9	52.9

*PCV* phenotypic coefficient of variation (%), *GCV* genotypic coefficient of variation (%), *H*^*2*^ broad-sense heritability (%), *GA* genetic advance, *GAM* genetic advance as percentage of the mean (%), *Max* maximum, *Min* minimum, *SEM* standard error of the mean.

* , ** and *** represent significant at *p* < 0.05, *p* < 0.01, and *p* < 0.001, respectively.

North Moroccan ecotypes were shorter and less leafy than Pakistani ones (108.5 vs. 145.0 cm and 7 vs. 12, for plant height and leaves number, respectively)^45^. Also, despite the lower leaf length and width that are responsible for photosynthesis, compared to ecotypes from Benin (31.7 vs. 76.4 cm and 3.4 vs. 7.5, respectively), the mean grain yield of the ecotypes (3.5 T/ha) was in the range of the value reported for traditional sorghum cultivars under restricted water conditions^46^. Additionally, the harvest index was higher compared to several Ethiopian landraces^47,48^, which implies that Moroccan ecotypes were selected by farmers for grain production at the expense of fodder production. Grains per plant (1473) and thousand seed weight (22.5 g) were in the range of values for the Ethiopian sorgho^49^. The days to flowering (82) were in the range of values reported in Ethiopia^47^. However, the ecotypes in the present study were earlier to reach maturity, which lowered the grain filling period compared to their study (31 vs. 55 days)^47^. The stem diameter in the present study was lower than other sorghum ecotypes^50,51^. It could be interesting from a bromatological perspective as it was reported to be negatively correlated to digestibility^52^. Low stem diameter was reported to be correlated to low resistance for lodging^53^. However, despite the low stem diameter, the ecotypes of the present study were lodging resistant (82%), probably due to their short height, explained by a selection of grains rather than fodder. *Bromatological characterization.* The choice of the best ecotypes to use in a particular region or a selection program depends not only on their productivity. The nutritive value plays a crucial role in supporting plant breeding programs. The long-term objective of the present study is to develop grain cultivars with high nutrient value since sorghum grains are principally used as feed in Northern Morocco. Moreover, using the straw after harvest could add extra interest. At the maturity stage, except for the grain DM content, the difference between the ecotypes was highly significant (p < 0.001) for all the bromatological parameters analyzed for the three parts of the plants (grains, leaves, and stems) (Table 4, Supplementary Tables 4, 5 and 6﻿). The bromatological parameters showed weaker variation than agro-morphological parameters. For sorghum grains, high GCV and PCV were found for ADF, ADL, CF, phenols, tannins (CT, TT), antioxidant activities (DPPH, FRAP), and CP digestibility. However, except for DM, NFE, and true digestibility, high heritability coupled with high GAM was found for all the parameters. It suggests that the selection based on these parameters will improve the nutritive value of the grains of the selected cultivars. For the leaves, only CP and Ash had high GCV and PCV. However, most parameters, including ADF, ADL, CF, EE, Ash, ME, and OM digestibility had high heritability coupled with high GAM. It testifies that improving the nutritive value of the sorghum leaves at maturity is also possible through selection. For the stems, only EE, CP, and ME showed high GCV and PCV, and except for DM, NDF, ADF, NFE, and true digestibility, all the other parameters had high heritability coupled with high GAM, suggesting that the stem nutritive value could also be improved through selection*.*

**Table 4:** Descriptive statistics and genetic parameters of bromatological traits in grains, leaves, and stems of 21 sorghum ecotypes studied in 2019 in Northern Morocco.

Trait	Max	Min	Mean	SEM	GCV	PCV	H^2^	GA	GAM
Grains									
Dry matter (DM, %)	89.6	81.3	85.9	0.7	0.6	1.5	13.6	0.4	0.4
Neutral detergent fiber (% DM)	37.1	16.4	26.0***	1.1	18.2	19.7	85.3	9.0	34.6
Acid detergent fiber (% DM)	15.4	5.2	6.7***	0.5	25.3	26.8	89.0	4.8	49.2
Acid detergent lignin (% DM)	10.1	2.9	5.5***	0.3	34.1	35.4	93.1	3.7	67.9
Crude fiber (% DM)	6.1	1.1	2.9***	0.2	44.0	45.4	93.9	2.6	87.8
Ether extract (% DM)	6.8	2.7	4.5***	0.3	18.6	21.7	73.3	1.5	32.8
Ash (% DM)	2.4	1.0	1.6***	0.1	19.7	20.3	93.8	0.6	39.2
Crude protein (% DM)	19.8	7.1	10.5***	0.7	14.0	18.2	58.8	2.3	22.1
Nitrogen-free extract (% DM)	84.5	58.0	80.3***	1.7	2.6	4.4	34.9	2.5	3.2
In vitro enzymatic organic matter digestibility (% DM)	96.9	60.3	81.4***	1.5	10.5	11.0	91.6	16.8	20.7
In vitro true digestibility (% DM)	98.7	79.1	92.2***	1.1	4.4	4.9	81.4	7.5	8.2
In vitro enzymatic crude protein digestibility (% DM)	53.3	22.0	35.3***	1.2	27.0	27.1	99.4	19.6	55.5
Metabolizable energy (MJ/kg DM)	13.8	8.1	11.4***	0.3	11.9	12.8	86.1	2.6	22.7
Total phenols (mg TAE/g DM)	180.8	49.4	110.2***	0.5	35.4	36.6	93.8	77.9	70.7
Condensed tannins (mg TAE/g DM)	9.1	2.5	5.0***	0.3	47.1	47.3	99.1	4.8	96.6
Total tannins (mg TAE/g DM)	12.1	2.8	6.0***	0.3	43.8	44.3	97.5	5.3	89.0
DPPH (IC50; µg/mL)	50.6	17.6	72.7***	0.0	39.8	40.0	99.2	59.4	81.7
FRAP (mg F_e_SO_4_/g DM)	27.6	12.0	17.1***	0.6	29.0	29.1	99.0	10.1	59.3
Leaves									
Dry matter (DM, %)	93.2	81.4	87.7***	1.4	2.3	3.5	42.7	2.7	3.1
Neutral detergent fiber (% DM)	75.8	55.5	65.5***	1.6	4.8	6.3	57.5	4.9	7.5
Acid detergent fiber (% DM)	49.0	21.0	36.9***	0.6	15.4	15.6	97.3	11.6	31.3
Acid detergent lignin (% DM)	33.0	13.0	25.5***	0.5	18.1	18.4	96.8	9.3	36.7
Crude fiber (% DM)	40.6	24.6	31.4***	0.5	13.0	13.3	95.6	8.2	26.1
Ether extract (% DM)	3.9	2.0	2.7***	0.1	18.2	18.7	95.0	1.0	36.6
Ash (% DM)	17.1	5.7	8.9***	0.7	21.8	25.5	72.8	3.4	38.2
Crude protein (% DM)	12.3	4.0	7.1***	0.3	28.6	29.5	93.8	4.0	57.0
Nitrogen-free extract (% DM)	61.1	37.0	49.9***	0.9	9.8	10.3	90.6	9.6	19.1
In vitro enzymatic organic matter digestibility (% DM)	50.3	28.5	40.9***	1.0	11.4	12.1	89.0	9.1	22.2
In vitro true digestibility (% DM)	82.9	61.8	72.8***	1.5	5.0	6.1	67.0	6.1	8.4
Metabolizable energy (MJ/kg DM)	6.5	1.7	4.1***	0.3	17.1	20.8	67.8	1.2	29.1
Stems									
Dry matter (DM, %)	95.8	83.0	88.8***	1.0	3.0	3.6	70.4	4.6	5.2
Neutral detergent fiber (% DM)	89.6	57.4	71.8***	3.0	6.9	10.0	46.8	7.0	9.7
Acid detergent fiber (% DM)	57.0	34.4	45.3***	2.1	7.2	10.7	45.3	4.5	10.0
Acid detergent lignin (% DM)	38.2	20.3	27.8***	0.7	13.9	14.6	91.6	7.6	27.5
Crude fiber (% DM)	49.9	31.4	40.4***	1.1	11.0	11.9	85.0	8.4	20.8
Ether extract (% DM)	1.8	0.6	1.1***	0.0	22.9	23.8	93.0	52.1	45.5
Ash (% DM)	8.3	2.9	5.1***	0.5	17.8	25.0	50.5	1.3	26.0
Crude protein (% DM)	3.8	1.0	2.4***	0.1	26.1	27.8	87.8	1.2	50.3
Nitrogen-free extract (% DM)	60.0	41.2	51.0***	1.2	8.8	9.6	83.1	8.4	16.5
In vitro enzymatic organic matter digestibility (% DM)	31.0	15.1	24.7***	0.9	14.0	15.4	82.5	6.5	26.1
In vitro true digestibility (% DM)	82.9	49.6	60.8***	2.4	10.4	12.5	69.0	10.8	17.8
Metabolizable energy (MJ/kg DM)	4.4	1.5	2.8***	0.2	23.8	27.6	74.4	1.2	42.2

*PCV* phenotypic coefficients of variation (%), *GCV* genotypic coefficients of variation (%), *H*^*2*^ broad-sense heritability (%), *GA* genetic advance, *GAM* genetic advance as percentage of the mean (%), *Max* maximum, *Min* minimum, *SEM* standard error of the mean.

*, ** and *** represent significant at *p* < 0.05, *p* < 0.01, and *p* < 0.001, respectively.

The grains' CP, EE, and Ash (10.5, 4.5, and 1.6% DM, respectively) were in the range of values in the Mediterranean area^56^. However, NDF values were higher than values reported in the USA (26.0 vs. 16.9% DM)^54^. Probably because the traditional Moroccan ecotypes contain a thick pericarp characterized by a high fiber quantity^55^. Despite this high NDF content, OM and true digestibilities (81.4 and 92.2%) were high but in the range of values reported for North-American ecotypes^56^. The leaf NDF and ADF values (65.5 and 36.9% DM, respectively) were in the range of values reported for USA hybrids^57^. According to Kamal et al.^58^, low fiber content is related to the leaf stay-green character, which was observed in almost 70% of the ecotypes. However, the stem NDF and ADF values (71.8 and 45.3% DM, respectively) were higher than the ones reported by the same authors^57^. These two parameters, in addition to NFE, CF, EE, and CP (51.0, 40.4, 1.1, and 2.4% DM, respectively), were in the range of values reported with Ethiopian ecotypes^59^. However, ADL content was higher (27.8 vs. 6% DM) and explained the lower stem ME for the ecotypes in the present study (2.8 vs. 7.1 MJ/kg DM). This higher stem ADL content could also strengthen the lodging resistance.

The sorghum grains and straw (leaves and stems) have different nutritive values and can be used for different purposes. The grains can be a good source of energy (11.4 MJ/kg DM for grains vs. 4.1 and 2.8 MJ/kg DM for leaves and stems, respectively). Alternatively, the straw can be a good source of fibers (higher NDF (26% DM for grains vs. 65.5 and 71.8% DM for leaves and stems, respectively), ADF (6.7% DM for grains vs. 36.9 and 45.3% DM for leaves and stems, respectively) and ADL (5.5% DM for grains vs. 25.5 and 27.8% DM for leaves and stems, respectively), but it was negatively reflected into a lower digestibility (81.4% DM for grains vs. 40.9 and 24.7% DM for leaves and stems respectively). The grains and leaves had high protein content (10.5 and 7.1% DM, respectively), while the stems had lower protein content (2.4% DM). Manifestly, the leaves were more nutritious compared to the stems. Similar results were reported for corn and pearl millet ^60,61^.

Total phenols of the grains (110.2 mg TAE/g DM) were lower than values reported for other Moroccan ecotypes^11^, probably due to the differences in the collected ecotypes. Their ecotypes grains were darker (brown and light brown) than present grains, where almost half of the grains were white, phenol contents being positively correlated to the darkness^62^. Condensed tannin content (5 mg TAE/g DM) was lower than values reported for type II and type III sorghum grains rich in phenols and tannins, which confirms that most of the ecotypes present in this study probably belong to type I sorghum grains^62^. Antioxidant activity (DPPH and FRAP) values were in the range of values reported by Kumari et al*.*^6^ for different sorghum ecotypes.

#### Correlation analysis

**Figure 2:**
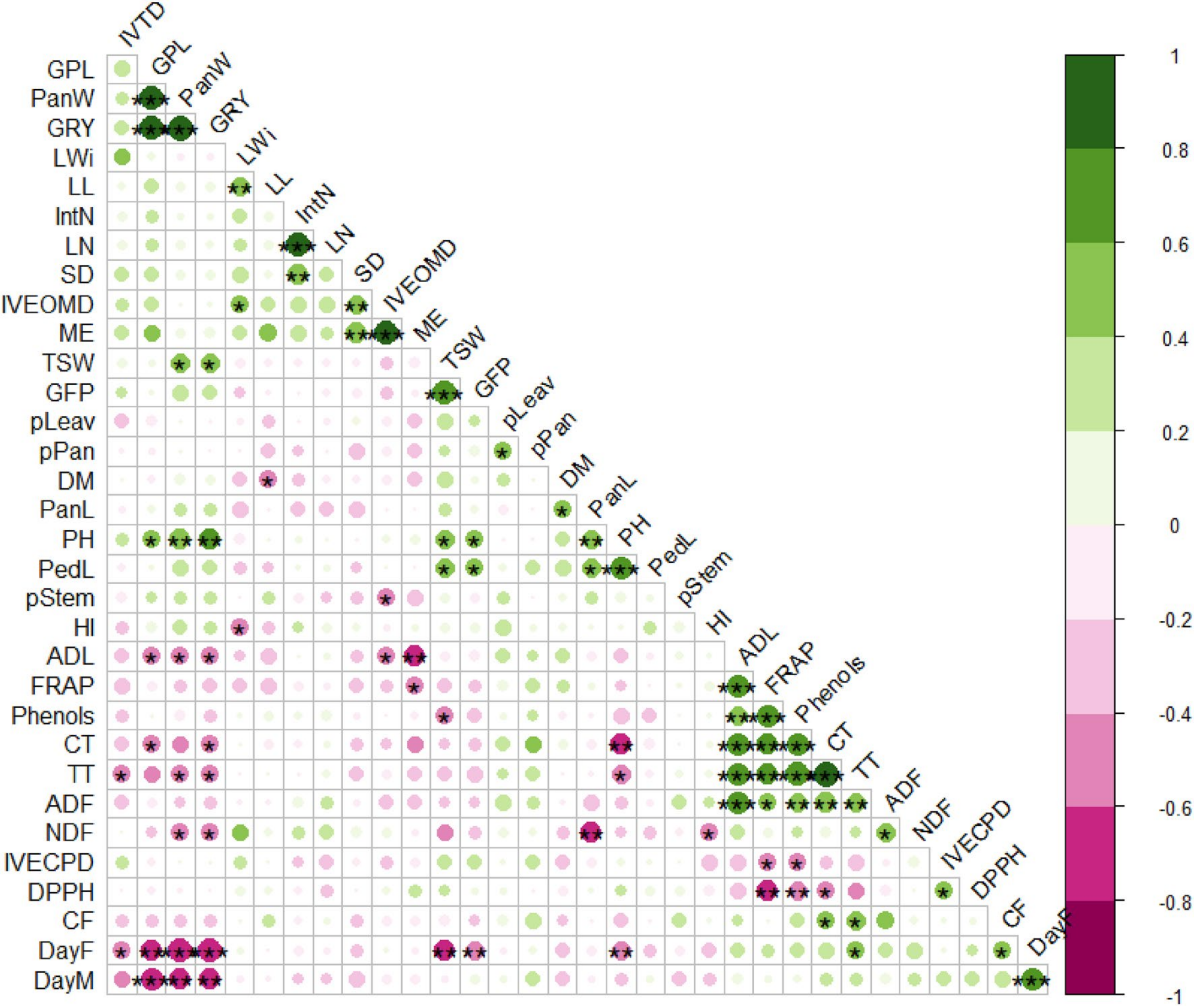
The correlation matrix of agro-morphological and bromatological traits evaluated in 21 sorghum ecotypes studied in 2019 in Northern Morocco. The color intensity and the size of the circle are proportional to the correlation coefficients. On the right side of the correlogram, the legend color shows the correlation coefficients and the corresponding colors. Positive correlations are displayed in green and negative correlations in purple color. *TSW* thousand seed weight (g), *GFP* grains filling period (days), *PanL* panicle length (cm), *PH* plant height (cm), *PedL* peduncle length (cm), *pStem* % of stems (%), *HI* harvest index (%), *CP* crude protein (% DM), *GPL* grains per plant, *PanW* panicle weight (g), *GRY* grains yield (T/ha), *CF* crude fiber (% DM), *DayF* days to flowering (days), *DayM* days to maturity (days), *pLeav* perventage of leaves (%), *pPan* percentage of panicle (%), *IVTD *in vitro true digestibility (%), *SD* stem diameter (cm), *IVEOMD *in vitro enzymatic organic matter digestibility (%), *ME* metabolizable energy (MJ/kg DM), *LWi* leaf width (cm), *LL* leaf length (cm), *IntN* internodes number, *LN* leaves number, *DPPH* 2,2-diphenyl-1-picrylhydrazyl (DPPH) scavenging activity (IC50, µg/mL), *FRAP* Ferric reducing ability of plasma (mg FeSO_4_/g DM), *Phenols* total phenols (mg TAE/g DM), *CT* condensed tannins (mg TAE/g DM), *TT* total tannins (mg TAE/g DM).

The knowledge of the association between yield, yield components, and bromatological parameters of the grains can help in the simultaneous selection of traits of interest for Moroccan sorghum crop improvement. The correlation matrix is reported in Fig. 2. Only significant (*p* < 0.05) correlations are discussed. As expected, positive correlations were found between grains per plant and grain yield and between thousand seed and panicle weights. Some negative correlations were reported between grain yield and days to flowering and to maturity, which could be explained by a drought resistance strategy of the ecotypes**.** The negative correlations of grain yield with NDF, ADL, CT, and TT were interesting and showed that farmers had selected grain high-yielding cultivars but also low fiber and tannin contents in their grains. Contrary to several studies that reported a negative association between grain yield and plant height^48^, plant height in the present study positively correlated with yield components, including grains per plant, grain yield, thousand seed weight, and panicle weight, indicating that the selection for higher plants could improve grain yield of the selected ecotypes. The strong positive correlation between thousand seed weight and grain filling period confirmed that a long maturation period could favor a good grain filling. Moreover, significant positive correlations were reported for leaf length and width with OM digestibility of the grains, possibly due to enhanced photosynthesis. The stem diameter also had positive correlations with grain OM digestibility and ME, showing that the breeders selected for high stem diameter (probably to protect the plants from lodging^53^) and also for grain nutritive value. Phenols, tannins, and antioxidant activities were positively correlated with ADF. Several authors reported the antioxidant activities of the phenols and tannins^63^. The higher antioxidant activity was observed in the “darkest” grains in the present study, these grains having the thickest pericarp and thus the highest ADF content. ADF had positive correlations with NDF and ADL, as also reported in other studies^46^. In grains, true digestibility was negatively correlated to TT, and CP digestibility was negatively correlated to Phenols. It could be explained by some grains' high phenol content (up to 18%) and its negative impact on the rumen microbiota^64^.

#### Principal component analysis

**Figure 3:**
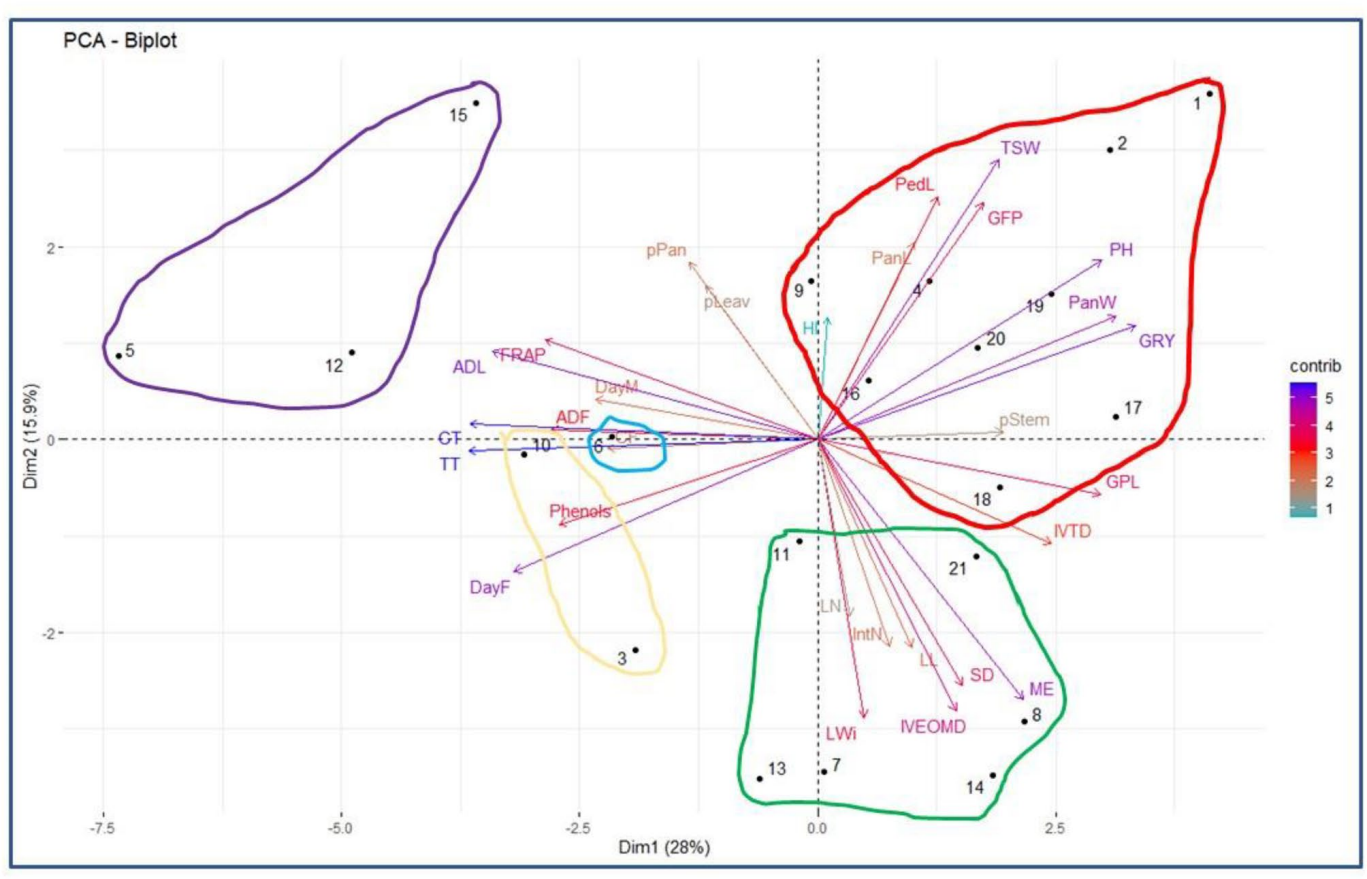
Graph of the variables and individuals of the principal component analysis. *TSW* thousand seed weight (g), *GFP* grain filling period (days), *PanL* panicle length (cm), *PH* plant height (cm), *PedL* peduncle length (cm), *pStem*, % of stems (%), *HI* harvest index (%), *CP* crude protein (% DM), *GPL* grains per plant, *PanW* panicle weight (g), *GRY* grain yield (T/ha), *CF* crude fiber (% DM), *DayF* days to flowering (days), *DayM* days to maturity (days), *pLeav* % of leaves (%), *pPan* % of panicle (%), *IVTD *in vitro true digestibility (%), *SD* stem diameter (cm), *IVEOMD *in vitro enzymatic organic matter digestibility (%), *ME* metabolizable energy (MJ/kg DM), *LWi* leaf width (cm), *LL* leaf length (cm), *IntN* internodes number, *LN* leaves number, *DPPH* 2,2-diphenyl-1-picrylhydrazyl (DPPH) scavenging activity (IC50, µg/mL), *FRAP* Ferric reducing ability of plasma *(*mg FeSO_4_/g DM), *Phenols* total phenols (mg TAE/g DM), *CT* condensed tannins (mg TAE/g DM), *TT* total tannins (mg TAE/g DM). Five clusters were determined by the cluster heatmap analysis and represented via the five colored circles (red, green, purple, yellow, and blue) on this figure.

The principal component analysis was conducted to cluster ecotypes based on parameters that participate mostly in the variation. The first three components explained 56.46% of the variability. Figure 3 represents the distribution of variables and individuals in the first two dimensions. The first component (PC1) participated with 28.0% in the variability. It was positively correlated to grains per plant and grain yield, panicle weight, and plant height and was negatively correlated to ADL, ADF, tannins, FRAP activity, Phenols, and days to flowering and maturity. The second component (PC2) explained 15.9% of the variability. It was positively correlated to thousand seed weight, peduncle length, and grain filling period and was negatively correlated to leaf length and width, internodes number, stem diameter, ME, and OM digestibility. According to these correlations, the PC1 was a positive agronomical (yield) and negative bromatological (fibers and antioxidants) component, while the PC2 was a negative agronomical (plant morphology) and negative bromatological (ME and OM digestibility) component. The opposite distribution of yield components with antioxidant content and activity could indicate that although the concentration of phenolic compounds in the grains could increase the antioxidant capacity, it could also negatively affect the absorption of essential nutrients (Fe, Mn, and P) by the plant, which hinders physiological processes, and thus reduce the yield^62^.

#### Heatmap analysis

**Figure 4:**
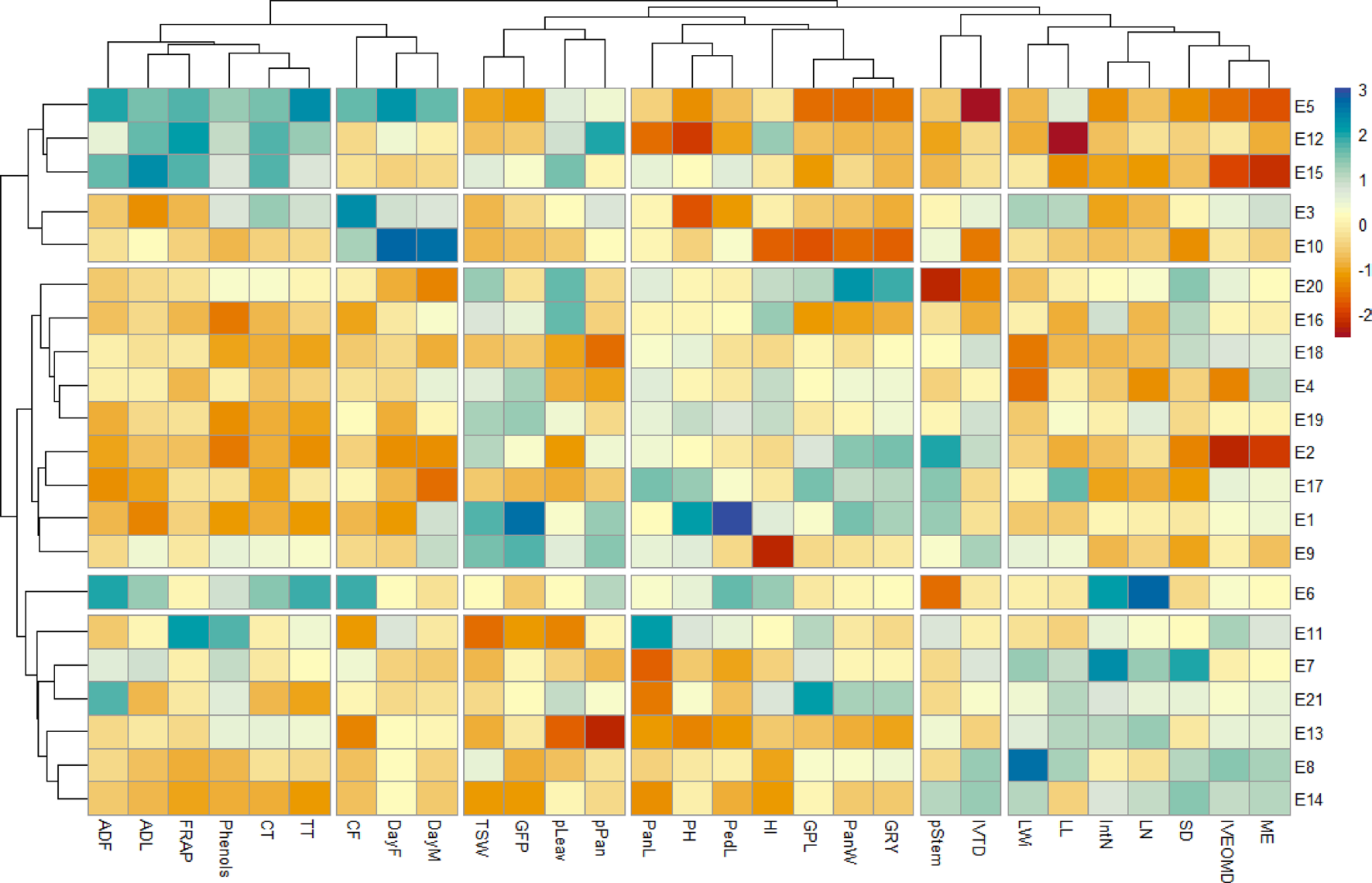
Cluster heatmap analysis of sorghum ecotypes’ responses to morpho-phenological, agronomic, and bromatological characterization. The heatmap plot describes the relative abundance of each sorghum ecotype (rows) within each trait (column). The color code (blue to dark red) displays the values of the parameters: blue color indicates high values while red color indicates low values. The dendrogram (on the left) shows the hierarchical clustering of sorghum ecotypes based on the Euclidian distance and Ward’s clustering method. *TSW* thousand seed weight (g), *GFP* grains filling period (days), *PanL* panicle length (cm), *PH* plant height (cm), *PedL* peduncle length (cm), *pStem* % of stems (%), *HI* harvest index (%), *CP* crude protein (% DM), *GPL* grains per plant, *PanW* panicle weight (g), *GRY* grain yield (T/ha), *CF* crude fiber (% DM), *DayF* days to flowering (days), *DayM* days to maturity (days), *pLeav* % of leaves (%), *pPan* % of panicle (%), *IVTD *in vitro true digestibility (%), *SD* stem diameter (cm), *IVEOMD *in vitro enzymatic organic matter digestibility (%), *ME* metabolizable energy (MJ/kg DM), *LWi* leaf width (cm), *LL* leaf length (cm), IntN internodes number, *LN* leaves number, *DPPH* 2,2-diphenyl-1-picrylhydrazyl (DPPH) scavenging activity (IC50, µg/mL), *FRAP* Ferric reducing ability of plasma (mg FeSO_4_/g DM), *Phenol*s total phenols (mg TAE/g DM), *CT* condensed tannins (mg TAE/g DM), *TT* total tannins (mg TAE/g DM).

A heatmap was conducted to cluster the ecotypes based on the agro-morpho-phenological and bromatological parameters (Fig. 4). The heatmap analysis structured the dendrogram on the left side of the figure according to sorghum ecotypes, and the second dendrogram at the top side of the figure showed the parameters that affected this distribution. The heatmap described five clusters, also represented in Fig. 3 by five colored circles. The explanatory variables were divided into six groups: the first group of high nutritive value and vegetative parameters (ME, OM digestibility, stem diameter, leaves number, length and width, and internodes number), the second group of true digestibility and percentage of stems, the third group of grain yield characteristics (grains per plant, grain yield, panicle length and weight, plant height, and peduncle length), the fourth group of grain weight (percentages of panicle and leaves, grain filling period, and thousand seed weight), the fifth group of phenology (days to flowering and to maturity, and CF) and the sixth group of fibers, and antioxidant content and activity (ADL, ADF, TT, CT, phenols, and FRAP). The first group's three ecotypes (E5, E12, and E15) were characterized by grains having low nutritive value and yield, and high content of fibers and phenols. The second group was similar to the first one except for the phenology (later ecotypes) and low fiber content. Opposite traits of the first group characterized the third group. High antioxidant factors and intermediate values for the other components characterized the only ecotype (E6) present in the fourth group. The fifth group differed by its higher nutritive value, more interesting vegetative parameters, and lower values for the other parameters.

According to this map, the choice of interesting ecotypes for feed should be based on the balance between plant and yield components, combined with high nutritive value. Ecotype 21 could be interesting as it combines high grain yield and high ME. Several studies highlight the importance of these dual-purpose genotypes^65^.

#### Mantel test

The Mantel test was conducted to link the environmental and agro-morphological data to bromatological values. The ecotypes were clustered into different groups irrespective of the region where they were collected. The Mantel test showed that no significant correlations were found between morpho-pheno-agronomic and bromatological parameters and geographical data (latitude and longitude) of the ecotype collection sites (r = -0.08, *p* = 0.82), nor with environmental data (r = -0.08, *p* = 0.81). However, as expected, geographic distance and environmental data were correlated (0.72, *p* < 0.001) for each collection site. The absence of significant correlation for the previous parameters could be due to the collection of ecotypes in a wider area covering all the northern region of Morocco or to seed exchange between farmers from the different regions. Consequently, choosing interesting ecotypes should be based on ecotypes level rather than geographical area.

## Conclusion

The present study showed that Moroccan ecotypes of *Sorghum bicolor* were highly variable for agro-morphological and bromatological parameters, with some resistance to drought. The grains presented interesting protein contents and metabolizable energy. The multivariate analysis distinguished five clusters based on agro-morphological, bromatological, and antioxidant activity. Selecting the better ecotypes could be based on ecotypes level rather than geographical area. This work being the first step, future multi-location trials across multiple cropping cycles are needed to confirm and strengthen the present results in order to improve sorghum selection and spread the best ecotypes. Moreover, conserving these local genes in a seed bank is useful since climate change and increasing recurrent droughts require maintaining a wide sorghum biodiversity bank.

### Supplementary Information

Supplementary Information.

## Data Availability

All data generated or analyzed during this study are included or specified in this published article.
