# Supplementary material for: Ecological, morpho-agronomical, and bromatological assessment of sorghum ecotypes in Northern Morocco
Source: Sci Rep. 2023 Sep 20;13:15548. doi: 10.1038/s41598-023-41565-9 (PMC10511723; doi:10.1038/s41598-023-41565-9)
Supplement: Supplementary file 1 — Supplementary Information. [file 41598_2023_41565_MOESM1_ESM.docx]

**Ecological, morpho-agronomical, and bromatological assessment of sorghum ecotypes in northern Morocco**

S. Boukrouh^1,2*^, A. Noutfia^2^, N. Moula^1^, C. Avril^3^, J. Louvieaux^3^, J.L. Hornick^1^, J.F. Cabaraux^1*^, M. Chentouf^2^

^1^ Department of Veterinary Management of Animal Resources, FARAH center, Faculty of Veterinary Medicine, University of Liège, 4000 Liège, Belgium

^2^ Regional Center of Agricultural Research of Tangier, National Institute of Agricultural Research, 10090 Rabat, Morocco

^3^ Haute École Provinciale de Hainaut Condorcet, agronomy category, 7800 Ath, Belgium

Corresponding authors: [s.boukrouh@uliege.be](mailto:s.boukrouh@uliege.be) , [jfcabaraux@uliege.be](mailto:jfcabaraux@uliege.be)

**Supplementary information**

**Table of content**

**Supplementary Table 1.** Voucher specimen entries for the 21 sorghum ecotypes in northern Morocco.

**Supplementary Table 2.** Climatic and physico-chemical characteristics of the soils of the 21 collection farms and the experimental cultivation site in northern Morocco.

**Supplementary Figure.** Representative panicle of each ecotype of the 21 ecotypes collected in northern Morocco.

**Supplementary Table 3.** Values of the 20 quantitative agro-morphological traits for the 21 sorghum studied ecotypes at the maturity stage.

**Supplementary Table 4.** Values of the 18 quantitative bromatological traits of grains of the 21 sorghum studied ecotypes at the maturity stage.

**Supplementary Table 5.** Values of the 12 quantitative bromatological traits of leaves of the 21 sorghum studied ecotypes at the maturity stage.

**Supplementary Table 6.** Values of the 12 quantitative bromatological traits of stems of the 21 sorghum studied ecotypes at the maturity stage.

| **Sites** | **Collection date** | **Latitude** | **Longitude** | **Elevation (m)** | **Commune** | **Caïdat** | **Cercle** | **Province/ Préfecture** | **Name of the botanist** | **Gene bank storage** |
| --- | --- | --- | --- | --- | --- | --- | --- | --- | --- | --- |
| 1 | 04/09/2018 | 34°40'36" | 4°36'24" | 650 | Khlalfa | Bouhouda | Taounate | Taounate | Researchers of INRA of Tangier | Gene Bank of INRA Morocco |
| 2 | 13/09/2018 | 35°15'32,37" | 5°58'52,23" | 80 | Rissana Chamalia | Rissana | Oued El Makhazine | Larache |  |  |
| 3 | 04/09/2018 | 34°45'26" | 5°06'04" | 323.5 | Tabouda | Beni Zeroual | Ghafsai | Taounate |  |  |
| 4 | 13/09/2018 | 35°27'1,17 | 5°53'21,16 | 68 | Lkhaloua | Gharbia | Assilah | Tanger Assilah |  |  |
| 5 | 05/09/2018 | 35°02'27" | 5°10'26" | 943 | Bni Salah | Bab Taza | Bab Taza | Chefchaouen |  |  |
| 6 | 05/09/2018 | 35° 15′ 27″ | 5° 20′ 27" | 521.5 | Kebhach | Bni Hassan | Tétouan | Tetouan |  |  |
| 7 | 13/09/2018 | 35°22'54,38" | 5°55'48,61" | 69 | Sidi El Yamani | Sidi El Yamani | Assilah | Tanger Assilah |  |  |
| 8 | 05/09/2018 | 34°52'22" | 5°32'18" | 709 | Bni Quolla | Bni Quolla | Moqrissat | Ouezzane |  |  |
| 9 | 13/09/2018 | 35°25'17,21 | 5°54'38,43 | 68 | Ayacha | Ayacha | Moulay Abdeslam-Ben Mchich | Tanger Assilah |  |  |
| 10 | 13/09/2018 | 35°22'54,38 | 5°55'48,61 | 69 | Sidi Lyamani | Sidi El Yamani | Assilah | Tanger Assilah |  |  |
| 11 | 13/09/2018 | 35°20'40,27" | 5°57'31,03" | 78 | Sidi Lyamani | Sidi Lyamani | Assilah | Tanger Assilah |  |  |
| 12 | 05/09/2018 | 35°6'10,46" | 5°27'31,5" | 304 | Tanaqoub | Tanaqoub | Bab taza | Chefchaouen |  |  |
| 13 | 05/09/2018 | 34°49'44" | 5°32'47" | 262 | Bni Quolla | Bni Quolla | Moqrissat | Ouezzane |  |  |
| 14 | 04/09/2018 | 34°41'54" | 5°00'17" | 558 | Sidi Yahya Beni zeroual | Beni Zeroual | Ghafsai | Tanounate |  |  |
| 15 | 10/10/2018 | 35°32'33" | 5°32'48" | 358 | Ain lahcen | Ain Lahcen | Jebala | Tétouan |  |  |
| 16 | 05/09/2018 | 34°30'26" | 4°46'17" | 254 | Mezraoua | Mezraoua | Taounate | Taounate |  |  |
| 17 | 05/09/2018 | 35°22'57,34" | 5°55'59,09" | 69 | Sidi El Yamani | Sidi El Yamani | Assilah | Tanger Assilah |  |  |
| 18 | 04/09/2018 | 34°40'36" | 4°36'24" | 644.5 | Khlalfa | Bouhouda | Taounate | Taounate |  |  |
| 19 | 13/09/2018 | 35°31'47,68" | 5°57'39,16" | 26 | Aquouass Briech | Gharbia | Assilah | Tanger Assilah |  |  |
| 20 | 10/10/2018 | 35°32'29" | 5°33'01" | 413 | Ain lahcen | Ain Lahcen | Jebala | Tétouan |  |  |
| 21 | 10/10/2018 | 35°06'06" | 5°18'383 | 540 | Dardara | Tanakoub | Chefchaouen | Chefchaouen |  |  |

**Supplementary Table 1.** Voucher specimen entries for the 21 sorghum ecotypes in Northern Morocco.

|  | **Horizon** | **pH water** | **pH KCl** | **EC** | **OM** | **CaCO3** | **Hum** | **N** | **C/N** | **P** | **K** | **Clay** | **Fine Silt** | **Coarse Sand** | **Fine Sand** | **Coarse Silt** |
| --- | --- | --- | --- | --- | --- | --- | --- | --- | --- | --- | --- | --- | --- | --- | --- | --- |
| **1** | 0-20 cm | 8.3 | 7.5 | 11.4 | 2.5 | 2.2 | 6.4 | 0.2 | 8.9 | 20.4 | 4.3 | 47.7 | 10.6 | 0.4 | 0.5 | 38.6 |
| **1** | 10-20cm | 8.6 | 7.6 | 9.4 | 3.1 | 2.3 | 6.3 | 0.2 | 11.2 | 20.3 | 3.8 | 47.7 | 10.6 | 0.4 | 0.4 | 38.0 |
| **2** | 0-20 cm | 8.6 | 8.0 | 13.6 | 2.5 | 1.0 | 6.6 | 0.2 | 8.0 | 109.6 | 4.6 | 0.0 | 18.0 | 0.7 | 1.0 | 80.3 |
| **2** | 10-20cm | 8.5 | 8.3 | 16.0 | 4.0 | 10.4 | 6.2 | 0.2 | 12.9 | 94.6 | 4.1 | 0.0 | 18.0 | 0.8 | 1.1 | 80.2 |
| **3** | 0-20 cm | 8.4 | 7.5 | 23.1 | 4.1 | 4.2 | 5.5 | 0.2 | 14.9 | 20.3 | 22.2 | 52.7 | 2.5 | 0.6 | 2.3 | 41.9 |
| **3** | 10-20cm | 8.5 | 7.6 | 20.3 | 3.2 | 4.2 | 5.2 | 0.2 | 12.4 | 20.4 | 24.6 | 60.7 | 2.0 | 0.5 | 2.3 | 34.5 |
| **4** | 0-20 cm | 8.7 | 8.4 | 16.4 | 1.7 | 6.2 | 5.3 | 0.1 | 6.8 | 153 | 37.2 | 65.8 | 10.0 | 0.2 | 0.2 | 23.8 |
| **4** | 10-20cm | 8.6 | 8.5 | 16.2 | 1.5 | 6.0 | 5.1 | 0.2 | 5.8 | 144 | 31.6 | 62.2 | 10.0 | 0.2 | 0.2 | 27.4 |
| **5** | 0-20 cm | 8.4 | 7.5 | 42.9 | 3.5 | 2.3 | 6.6 | 0.2 | 9.7 | 20.5 | 19.3 | 42.8 | 10.7 | 0.3 | 3.5 | 40.4 |
| **5** | 10-20cm | 8.5 | 7.6 | 37.9 | 3.7 | 2.0 | 6.6 | 0.2 | 10.8 | 20.4 | 20.8 | 37.5 | 5.4 | 0.3 | 3.8 | 51.2 |
| **6** | 0-20 cm | 8.5 | 7.4 | 41.2 | 2.6 | 2.4 | 6.2 | 0.1 | 10.8 | 20.3 | 4.4 | 51.2 | 11.3 | 0.3 | 0.4 | 36.8 |
| **6** | 10-20cm | 8.3 | 7.5 | 35.0 | 2.3 | 2.3 | 6.2 | 0.2 | 8.9 | 20.3 | 4.6 | 50.4 | 12.7 | 0.3 | 0.4 | 36.1 |
| **7** | 0-20 cm | 7.9 | 7.4 | 53.1 | 4.2 | 1.4 | 3.5 | 0.2 | 12.7 | 20.5 | 6.0 | 30.1 | 5.2 | 3.6 | 6.4 | 52.6 |
| **7** | 10-20cm | 7.9 | 7.4 | 47.3 | 4.0 | 1.4 | 3.7 | 0.2 | 12.8 | 21.6 | 6.1 | 25.8 | 5.2 | 6.0 | 5.0 | 56.8 |
| **8** | 0-20 cm | 4.6 | 4.7 | 70.5 | 2.8 | 0.8 | 4.6 | 0.2 | 7.0 | 138 | 120.6 | 0.0 | 20.0 | 0.7 | 0.9 | 77.1 |
| **8** | 10-20cm | 4.3 | 4.6 | 76.1 | 1.4 | 1.4 | 7.1 | 0.1 | 7.6 | 57.2 | 150.7 | 0.0 | 20.0 | 0.7 | 0.9 | 78.4 |
| **9** | 0-20 cm | 8.7 | 8.3 | 51.2 | 1.9 | 0.9 | 5.2 | 0.2 | 6.2 | 131.4 | 194.5 | 1.0 | 25.0 | 16.0 | 28.0 | 30.0 |
| **9** | 10-20cm | 8.5 | 8.4 | 54.6 | 1.7 | 1.0 | 4.7 | 0.2 | 6.2 | 118.6 | 178.6 | 1.0 | 24.0 | 15.8 | 30.0 | 29.2 |
| **10** | 0-20 cm | 8.7 | 8.4 | 14.8 | 2.3 | 1.1 | 4.6 | 0.1 | 9.7 | 147 | 172.9 | 0.0 | 22.0 | 15.0 | 26.5 | 36.5 |
| **10** | 10-20cm | 8.1 | 7.8 | 12.2 | 2.1 | 1.0 | 3.6 | 0.1 | 8.8 | 134 | 157.4 | 0.0 | 19.0 | 15.0 | 27.0 | 39.0 |
| **11** | 0-20 cm | 7.4 | 7.3 | 73.5 | 2.0 | 1.2 | 3.6 | 0.1 | 8.3 | 143.5 | 180.8 | 0.0 | 20.0 | 14.7 | 25.9 | 39.5 |
| **11** | 10-20cm | 6.7 | 7.5 | 79.1 | 1.9 | 1.1 | 3.6 | 0.4 | 2.9 | 125.6 | 167.8 | 0.0 | 20.0 | 14.7 | 25.9 | 39.5 |
| **12** | 0-20 cm | 5.0 | 4.5 | 73.5 | 5.6 | 0.7 | 5.9 | 0.4 | 8.8 | 184.7 | 361.7 | 30.0 | 10.0 | 3.8 | 2.0 | 54.2 |
| **12** | 10-20cm | 5.0 | 4.4 | 71.8 | 3.4 | 0.8 | 5.9 | 0.3 | 6.1 | 64.4 | 422.0 | 30.0 | 10.0 | 3.8 | 2.0 | 54.2 |
| **13** | 0-20 cm | 8.8 | 8.8 | 54.6 | 0.6 | 7.0 | 5.8 | 0.1 | 3.0 | 50.3 | 211.0 | 0.0 | 20.0 | 6.5 | 1.0 | 72.5 |
| **13** | 10-20cm | 8.2 | 7.9 | 45.7 | 1.7 | 8.1 | 5.8 | 0.2 | 6.2 | 67.6 | 211.0 | 0.0 | 20.0 | 6.5 | 1.0 | 72.5 |
| **14** | 0-20 cm | 8.5 | 7.6 | 15.4 | 4.3 | 4.1 | 6.7 | 0.2 | 16.5 | 20.3 | 18.4 | 53.5 | 5.4 | 0.6 | 2.5 | 38.1 |
| **14** | 10-20cm | 8.4 | 7.6 | 14.3 | 3.4 | 4.1 | 6.6 | 0.2 | 12.4 | 20.4 | 20.6 | 58.9 | 0.0 | 0.5 | 2.5 | 38.2 |
| **15** | 0-20 cm | 8.3 | 8.5 | 72.6 | 2.3 | 2.1 | 5.5 | 0.1 | 13.6 | 132.8 | 124.6 | 25.0 | 8.0 | 4.2 | 0.5 | 62.3 |
| **15** | 10-20cm | 8.3 | 8.4 | 74.1 | 2.2 | 2.3 | 5.8 | 0.1 | 12.5 | 127.7 | 121.7 | 25.0 | 8.0 | 4.5 | 0.6 | 61.9 |
| **16** | 0-20 cm | 6.9 | 6.6 | 20.2 | 4.8 | 1.0 | 9.2 | 0.2 | 14.7 | 20.3 | 21.1 | 71.5 | 5.5 | 0.4 | 0.4 | 22.2 |
| **16** | 10-20cm | 7.7 | 6.6 | 19.8 | 5.7 | 1.0 | 9.2 | 0.2 | 17.3 | 20.3 | 22.9 | 77.0 | 0.0 | 0.4 | 0.4 | 22.2 |
| **17** | 0-20 cm | 8.2 | 7.9 | 53.3 | 1.2 | 0.8 | 4.3 | 0.1 | 11.8 | 59.2 | 241.1 | 10.0 | 10.0 | 19.2 | 8.3 | 52.6 |
| **17** | 10-20cm | 8.9 | 8.6 | 52.4 | 0.2 | 1.2 | 3.1 | 0.1 | 2.6 | 72.3 | 180.8 | 10.0 | 10.0 | 19.2 | 8.3 | 52.6 |
| **18** | 0-20 cm | 7.5 | 7.0 | 1.6 | 3.7 | 1.6 | 7.8 | 0.2 | 11.8 | 20.4 | 12.4 | 60.7 | 8.1 | 0.4 | 0.5 | 30.4 |
| **18** | 10-20cm | 8.2 | 7.1 | 1.4 | 4.4 | 1.6 | 7.2 | 0.2 | 12.7 | 20.4 | 13.6 | 63.8 | 5.4 | 0.4 | 0.4 | 29.9 |
| **19** | 0-20 cm | 8.4 | 7.4 | 13.1 | 4.5 | 2.3 | 9.5 | 0.2 | 15.3 | 20.4 | 25.5 | 82.5 | 0.0 | 0.2 | 0.1 | 17.3 |
| **19** | 10-20cm | 8.4 | 7.4 | 13.9 | 4.0 | 2.2 | 9.6 | 0.2 | 14.3 | 20.3 | 24.1 | 82.4 | 0.0 | 0.2 | 0.4 | 17.0 |
| **20** | 0-20 cm | 8.2 | 8.5 | 63.5 | 2.1 | 2.0 | 4.5 | 0.1 | 15.3 | 153.4 | 120.6 | 20.0 | 10.0 | 3.3 | 0.5 | 66.2 |
| **20** | 10-20cm | 8.2 | 8.3 | 64.9 | 1.9 | 1.9 | 4.6 | 0.1 | 18.5 | 141.7 | 118.6 | 19.0 | 10.0 | 3.4 | 0.4 | 67.2 |
| **21** | 0-20 cm | 8.0 | 7.8 | 77.2 | 5.1 | 1.1 | 6.9 | 0.2 | 13.6 | 116.7 | 10.7 | 0.0 | 21.0 | 0.7 | 0.9 | 77.4 |
| **21** | 10-20cm | 8.2 | 8.0 | 75.5 | 4.8 | 1.2 | 6.7 | 0.2 | 14.8 | 108.1 | 11.6 | 0.0 | 22.0 | 0.7 | 0.9 | 76.4 |

**Supplementary Table 2.** Climatic and physico-chemical characteristics of the soils of the 21 collection farms and the experimental cultivation site in northern Morocco. *EC* electrical conductivity (mS/m), *OM* organic matter (%), *CaCO3* limestone (%), *Hum* humidity (%), *N* nitrogen (%), *C/N* carbon to nitrogen ratio, *P* Available phosphorus (ppm), *K* Exchangeable potassium (ppm).

|   E1 |   E2 |   E3 |   E4 |   E5 |   E6 |   E7 |
| --- | --- | --- | --- | --- | --- | --- |
|   E7 |   E8 |   E9 |   E10 |   E11 |   E12 |   E13 |
|   E15 |   E16 |   E17 |   E18 |   E19 |   E20 |   E21 |

**Supplementary Figure 1.** Representative panicle of each ecotype of the 21 ecotypes (E1-21) collected in northern Morocco.

| **Ecotype** | **PH** | **INTN** | **LN** | **LL** | **LW** | **SD** | **pLEAV** | **pSTEM** | **pPAN** | **PedL** | **PanL** | **PanWi** | **PanW** | **DF** | **GRFP** | **DM** | **GRPL** | **GRY** | **HI** | **TSW** |
| --- | --- | --- | --- | --- | --- | --- | --- | --- | --- | --- | --- | --- | --- | --- | --- | --- | --- | --- | --- | --- |
| E1 | 161.0 | 7.6 | 6.7 | 28.9 | 3.1 | 0.8 | 8.3 | 5.2 | 52.7 | 78.9 | 18.8 | 9.7 | 66.8 | 67.7 | 54.0 | 121.7 | 1701.1 | 5662.4 | 60.8 | 32.6 |
| E2 | 114.7 | 6.8 | 6.5 | 27.3 | 3.2 | 0.6 | 9.7 | 5.9 | 57.6 | 34.1 | 19.7 | 7.5 | 65.3 | 66.0 | 34.0 | 100.0 | 1934.4 | 6316.9 | 47.1 | 28.7 |
| E3 | 62.4 | 6.5 | 6.0 | 37.1 | 3.9 | 0.8 | 11.5 | 18.1 | 42.0 | 20.5 | 17.8 | 5.3 | 25.7 | 92.3 | 28.7 | 121.0 | 1069.1 | 1968.2 | 52.2 | 17.6 |
| E4 | 111.3 | 6.9 | 5.6 | 31.5 | 2.7 | 0.8 | 11.8 | 14.1 | 37.1 | 34.1 | 20.9 | 9.6 | 46.5 | 76.7 | 41.7 | 118.3 | 1598.9 | 4369.8 | 65.7 | 26.1 |
| E5 | 75.9 | 6.3 | 6.1 | 34.6 | 3.0 | 0.6 | 5.2 | 21.3 | 36.6 | 26.7 | 15.4 | 3.0 | 10.5 | 109.0 | 21.3 | 130.3 | 460.8 | 849.8 | 49.2 | 16.2 |
| E6 | 121.4 | 9.7 | 9.1 | 31.3 | 3.4 | 0.8 | 4.9 | 7.5 | 27.9 | 59.9 | 19.4 | 7.3 | 42.0 | 84.0 | 26.7 | 110.7 | 1403.2 | 3887.4 | 71.5 | 23.8 |
| E7 | 94.5 | 10.0 | 7.9 | 36.5 | 3.9 | 1.1 | 8.6 | 12.6 | 37.9 | 21.4 | 9.5 | 5.7 | 40.0 | 75.0 | 32.0 | 107.0 | 1951.3 | 3611.2 | 44.4 | 17.6 |
| E8 | 104.4 | 7.5 | 6.5 | 37.6 | 4.4 | 1.0 | 11.9 | 12.3 | 38.4 | 35.8 | 15.3 | 7.5 | 45.5 | 84.3 | 23.7 | 108.0 | 1687.8 | 4361.5 | 36.6 | 25.3 |
| E9 | 125.4 | 6.7 | 6.3 | 33.9 | 3.6 | 0.7 | 10.0 | 15.8 | 44.2 | 31.5 | 20.0 | 6.7 | 35.5 | 76.3 | 46.7 | 123.0 | 1253.3 | 3332.8 | 19.6 | 31.6 |
| E10 | 95.4 | 6.9 | 6.2 | 29.0 | 3.3 | 0.6 | 4.5 | 16.4 | 44.5 | 40.6 | 18.2 | 3.7 | 10.4 | 115.3 | 25.3 | 140.7 | 298.4 | 587.3 | 27.4 | 17.7 |
| E11 | 128.9 | 8.1 | 6.9 | 29.5 | 3.3 | 0.9 | 8.0 | 9.5 | 47.9 | 42.9 | 27.8 | 6.8 | 36.3 | 90.3 | 21.7 | 112.0 | 2175.4 | 2838.4 | 53.9 | 12.9 |
| E12 | 55.5 | 6.9 | 6.5 | 20.0 | 3.0 | 0.7 | 5.2 | 12.3 | 32.6 | 21.7 | 9.9 | 5.5 | 23.8 | 86.7 | 26.3 | 113.0 | 1039.6 | 2055.1 | 70.4 | 18.1 |
| E13 | 73.0 | 8.7 | 7.9 | 36.9 | 3.6 | 0.8 | 9.4 | 17.0 | 45.3 | 19.5 | 12.3 | 5.8 | 23.5 | 84.0 | 30.0 | 114.0 | 997.0 | 1811.1 | 43.5 | 17.2 |
| E14 | 108.2 | 8.3 | 7.5 | 29.7 | 3.8 | 1.1 | 8.2 | 14.3 | 50.4 | 22.7 | 11.8 | 5.9 | 29.2 | 84.7 | 21.7 | 106.3 | 1532.7 | 2496.9 | 35.1 | 16.1 |
| E15 | 114.5 | 6.5 | 5.7 | 26.0 | 3.3 | 0.7 | 6.4 | 12.3 | 35.0 | 44.9 | 20.3 | 6.2 | 33.2 | 75.3 | 33.7 | 109.0 | 695.5 | 2156.4 | 49.5 | 26.1 |
| E16 | 110.4 | 8.5 | 6.0 | 27.5 | 3.4 | 1.0 | 5.7 | 12.8 | 39.7 | 38.0 | 17.9 | 6.1 | 20.7 | 80.0 | 35.7 | 115.7 | 706.1 | 1927.2 | 70.6 | 27.0 |
| E17 | 140.7 | 6.5 | 5.9 | 39.4 | 3.4 | 0.7 | 9.2 | 7.8 | 53.7 | 41.0 | 25.0 | 5.6 | 56.8 | 71.0 | 24.7 | 95.7 | 2461.4 | 5562.8 | 49.6 | 19.3 |
| E18 | 121.8 | 6.7 | 6.1 | 27.9 | 2.8 | 1.0 | 6.0 | 8.8 | 42.9 | 32.8 | 19.2 | 7.0 | 35.5 | 76.7 | 26.3 | 103.0 | 1514.3 | 3924.1 | 47.2 | 18.6 |
| E19 | 132.2 | 7.5 | 7.3 | 33.3 | 3.1 | 0.8 | 3.9 | 7.8 | 41.7 | 46.4 | 20.2 | 8.8 | 41.1 | 70.7 | 42.7 | 113.3 | 1399.8 | 4239.3 | 65.5 | 29.5 |
| E20 | 110.3 | 7.7 | 7.0 | 31.8 | 3.1 | 1.0 | 3.3 | 3.6 | 22.4 | 36.3 | 20.5 | 8.6 | 78.9 | 70.0 | 29.0 | 99.0 | 2187.9 | 6869.0 | 65.4 | 30.0 |
| E21 | 116.1 | 8.3 | 7.2 | 36.9 | 3.5 | 0.9 | 7.9 | 6.9 | 38.1 | 25.6 | 10.7 | 7.0 | 61.4 | 77.0 | 33.3 | 110.3 | 2863.3 | 5570.4 | 63.4 | 20.4 |

**Supplementary Table 3.** Values of the 20 quantitative agro-morphological traits for the 21 sorghum studied ecotypes at the maturity stage. *PH* plant height (cm), INTN internodes number, *LN* leaves number, *LL* leaves length (cm), *LW* leaves width (cm), *SD* stem diameter (cm), *pLeav* percentage of leaves (%), *pStem* percentage of stems (%), ), *pPan* percentage of panicle (%), *PedL* peduncle length (cm), *PanL* panicle length (cm), *PanWi* panicle width (cm), *PanW* panicle weight (g), *DayF* days to flowering (days), *GFP* grains filling period (days), *DayM* days to maturity (days), *GPL* grains per plant, *GRY* grains yield (T/ha), *HI* harvest index (%), *TSW* thousand seed weight (g).

| **Ecotype** | **DM** | **NDF** | **ADF** | **ADL** | **CF** | **EE** | **Ash** | **CP** | **NFE** | **IVEOMD** | **IVTD** | **ME** | **IVECPD** | **Phenols** | **CT** | **TT** | **DPPH** | **FRAP** |
| --- | --- | --- | --- | --- | --- | --- | --- | --- | --- | --- | --- | --- | --- | --- | --- | --- | --- | --- |
| **E1** | 85.9 | 25.0 | 7.8 | 3.0 | 1.9 | 5.6 | 1.8 | 9.3 | 81.3 | 83.8 | 91.3 | 12.0 | 47.2 | 62.3 | 2.7 | 3.1 | 74.1 | 14.6 |
| **E2** | 86.2 | 19.6 | 7.2 | 4.1 | 2.3 | 5.4 | 1.6 | 11.6 | 79.2 | 61.5 | 96.2 | 8.5 | 53.3 | 49.4 | 2.9 | 2.8 | 55.8 | 13.8 |
| **E3** | 85.2 | 22.4 | 8.2 | 3.0 | 5.9 | 4.3 | 1.4 | 10.1 | 78.4 | 86.2 | 94.6 | 12.6 | 43.5 | 139.7 | 8.0 | 8.4 | 63.5 | 12.8 |
| **E4** | 85.9 | 16.6 | 9.5 | 5.3 | 2.6 | 3.9 | 1.7 | 12.1 | 79.7 | 69.5 | 92.4 | 12.7 | 33.3 | 106.9 | 3.4 | 4.8 | 113.3 | 13.0 |
| **E5** | 84.8 | 33.3 | 14.5 | 8.3 | 5.1 | 4.5 | 2.1 | 8.2 | 80.1 | 67.6 | 81.6 | 8.9 | 36.5 | 163.2 | 8.6 | 12.1 | 55.8 | 25.9 |
| **E6** | 86.8 | 27.5 | 14.6 | 7.9 | 5.3 | 6.2 | 1.3 | 11.5 | 75.7 | 84.4 | 91.5 | 11.7 | 28.1 | 143.1 | 8.2 | 10.9 | 75.0 | 17.4 |
| **E7** | 84.6 | 32.2 | 11.3 | 7.1 | 3.5 | 4.4 | 2.3 | 11.1 | 78.8 | 81.0 | 95.3 | 11.6 | 31.4 | 149.4 | 4.7 | 6.4 | 37.3 | 16.7 |
| **E8** | 85.4 | 27.3 | 8.8 | 4.2 | 2.0 | 4.9 | 1.9 | 14.2 | 77.0 | 94.1 | 97.5 | 13.0 | 42.8 | 77.0 | 4.5 | 5.1 | 104.8 | 12.4 |
| **E9** | 86.1 | 29.7 | 8.7 | 6.2 | 2.5 | 4.6 | 1.6 | 9.2 | 82.1 | 80.7 | 97.0 | 10.4 | 47.2 | 133.0 | 5.8 | 6.9 | 80.9 | 16.4 |
| **E10** | 85.5 | 28.1 | 9.1 | 5.8 | 4.5 | 3.1 | 1.3 | 8.5 | 82.6 | 80.2 | 86.0 | 10.8 | 44.0 | 79.6 | 3.9 | 4.9 | 136.2 | 14.9 |
| **E11** | 86.6 | 22.3 | 8.1 | 5.7 | 1.4 | 3.1 | 1.3 | 12.3 | 81.9 | 92.1 | 92.1 | 12.4 | 22.5 | 180.7 | 4.9 | 7.1 | 28.9 | 27.6 |
| **E12** | 87.0 | 25.9 | 11.1 | 8.7 | 2.5 | 3.4 | 1.6 | 11.7 | 80.7 | 80.1 | 90.6 | 10.1 | 23.3 | 147.7 | 9.1 | 9.5 | 35.8 | 27.3 |
| **E13** | 85.4 | 27.5 | 8.7 | 5.2 | 1.2 | 6.0 | 1.2 | 8.5 | 83.2 | 86.3 | 90.3 | 12.1 | 25.1 | 129.9 | 6.2 | 7.0 | 32.8 | 15.3 |
| **E14** | 86.2 | 35.0 | 8.3 | 3.9 | 2.0 | 5.1 | 1.1 | 7.7 | 84.2 | 90.1 | 97.6 | 12.9 | 38.1 | 72.5 | 2.9 | 2.9 | 104.8 | 12.0 |
| **E15** | 87.5 | 27.5 | 13.8 | 9.7 | 2.6 | 4.5 | 1.6 | 10.7 | 80.6 | 64.9 | 91.2 | 8.3 | 31.9 | 140.0 | 9.0 | 7.9 | 50.0 | 25.5 |
| **E16** | 85.6 | 24.5 | 8.0 | 4.8 | 1.6 | 5.0 | 1.5 | 10.2 | 81.8 | 81.9 | 88.3 | 11.3 | 42.8 | 53.0 | 3.0 | 4.7 | 107.0 | 13.3 |
| **E17** | 85.6 | 22.1 | 6.6 | 3.4 | 3.1 | 3.3 | 1.7 | 9.1 | 82.8 | 86.1 | 90.6 | 11.9 | 23.6 | 101.4 | 2.5 | 5.5 | 86.6 | 15.7 |
| **E18** | 86.5 | 26.7 | 9.5 | 4.9 | 2.2 | 4.8 | 1.4 | 10.9 | 80.8 | 87.5 | 96.0 | 12.2 | 34.8 | 67.7 | 2.7 | 3.3 | 60.0 | 16.3 |
| **E19** | 87.3 | 21.2 | 7.5 | 4.8 | 3.2 | 3.9 | 1.3 | 10.9 | 80.6 | 82.4 | 96.0 | 11.5 | 26.3 | 58.7 | 2.7 | 3.4 | 73.1 | 15.7 |
| **E20** | 87.2 | 19.2 | 8.3 | 4.9 | 2.8 | 3.8 | 1.3 | 10.6 | 81.5 | 85.5 | 86.3 | 11.6 | 22.0 | 124.7 | 5.6 | 6.1 | 65.0 | 15.8 |
| **E21** | 86.2 | 31.3 | 14.0 | 4.0 | 3.0 | 5.1 | 1.9 | 12.0 | 78.1 | 84.0 | 93.6 | 12.2 | 43.7 | 134.2 | 3.0 | 3.2 | 86.5 | 16.5 |

**Supplementary Table 4.** Values of the 18 quantitative bromatological traits of grains of the 21 sorghum studied ecotypes at the maturity stage. *DM* Dry matter (%), *NDF* Neutral detergent fiber (% DM), *ADF* Acid detergent fiber (% DM), *ADL* Acid detergent lignin (% DM), *CF* Crude fiber (% DM), *EE* Ether extract (% DM), Ash (% DM), *CP* Crude protein (% DM), *NFE* Nitrogen-free extract (% DM), *IVEOMD* *in vitro* enzymatic organic matter digestibility (%), *IVTD* *in vitro* true digestibility (%), *ME* metabolizable energy (KJ/kg DM), *IVECPD in vitro* enzymatic organic matter digestibility (%), *Phenols* total phenols (mg TAE/g DM), *CT* condensed tannins (mg TAE/g DM), *TT* total tannins (mg TAE/g DM), *DPPH* 2,2-diphenyl-1-picrylhydrazyl (DPPH) scavenging activity (IC50, µg/mL), *FRAP* Ferric reducing ability of plasma (mg FeSO4/g DM).

| **Ecotype** | **DM** | **NDF** | **ADF** | **ADL** | **CF** | **EE** | **Ash** | **CP** | **NFE** | **IVEOMD** | **IVTD** | **ME** |
| --- | --- | --- | --- | --- | --- | --- | --- | --- | --- | --- | --- | --- |
| E1 | 84.0 | 68.5 | 44.0 | 29.7 | 26.5 | 2.2 | 7.2 | 6.3 | 57.8 | 42.3 | 72.4 | 3.9 |
| E2 | 85.3 | 66.8 | 39.2 | 30.7 | 32.7 | 3.6 | 7.7 | 5.1 | 50.9 | 41.7 | 75.4 | 3.5 |
| E3 | 89.3 | 63.8 | 36.9 | 28.7 | 25.5 | 2.3 | 10.8 | 8.1 | 53.3 | 39.7 | 73.1 | 4.2 |
| E4 | 90.7 | 65.5 | 36.3 | 26.7 | 28.8 | 2.9 | 9.1 | 8.3 | 51.0 | 42.1 | 74.5 | 3.9 |
| E5 | 85.8 | 63.5 | 34.8 | 29.6 | 28.6 | 3.7 | 7.6 | 8.1 | 52.0 | 44.1 | 78.5 | 4.1 |
| E6 | 87.2 | 65.6 | 39.8 | 25.9 | 29.5 | 3.2 | 10.5 | 4.6 | 52.2 | 49.0 | 74.2 | 4.5 |
| E7 | 91.6 | 66.4 | 32.6 | 27.3 | 25.9 | 2.4 | 7.1 | 6.2 | 58.4 | 40.6 | 73.0 | 4.0 |
| E8 | 86.9 | 63.8 | 38.5 | 27.7 | 39.1 | 2.5 | 16.2 | 4.7 | 37.4 | 44.0 | 74.0 | 3.8 |
| E9 | 88.1 | 70.3 | 35.9 | 22.5 | 29.8 | 2.5 | 7.9 | 6.3 | 53.4 | 39.8 | 71.1 | 4.3 |
| E10 | 83.9 | 61.6 | 36.7 | 27.6 | 39.2 | 2.4 | 7.5 | 6.1 | 44.8 | 42.8 | 75.1 | 4.2 |
| E11 | 91.7 | 65.9 | 21.8 | 13.8 | 35.7 | 2.5 | 8.8 | 5.2 | 47.9 | 32.3 | 63.8 | 3.1 |
| E12 | 89.2 | 69.1 | 39.3 | 32.2 | 30.1 | 2.0 | 9.0 | 5.7 | 53.2 | 40.5 | 73.6 | 3.8 |
| E13 | 85.7 | 63.6 | 31.9 | 21.4 | 28.0 | 2.9 | 8.0 | 11.8 | 49.4 | 47.4 | 73.9 | 5.9 |
| E14 | 87.6 | 65.8 | 45.3 | 23.0 | 35.9 | 3.1 | 8.0 | 5.5 | 47.5 | 41.2 | 75.9 | 4.1 |
| E15 | 88.6 | 64.5 | 36.6 | 26.4 | 37.1 | 2.7 | 10.8 | 7.0 | 42.5 | 43.7 | 78.8 | 4.4 |
| E16 | 91.5 | 62.0 | 30.0 | 20.3 | 29.3 | 2.4 | 9.0 | 6.1 | 53.2 | 42.8 | 77.4 | 4.3 |
| E17 | 86.7 | 71.5 | 47.7 | 32.2 | 32.9 | 2.2 | 7.1 | 6.0 | 51.8 | 31.2 | 67.0 | 1.8 |
| E18 | 87.5 | 56.2 | 43.8 | 20.9 | 29.8 | 3.8 | 7.8 | 10.7 | 47.8 | 44.4 | 70.4 | 4.9 |
| E19 | 88.7 | 68.7 | 35.0 | 20.0 | 32.5 | 3.1 | 7.7 | 9.4 | 47.3 | 30.1 | 66.7 | 3.7 |
| E20 | 84.4 | 63.0 | 35.4 | 23.4 | 33.5 | 2.4 | 9.6 | 10.4 | 44.2 | 38.2 | 68.6 | 4.5 |
| E21 | 86.3 | 69.5 | 33.3 | 24.6 | 28.9 | 2.7 | 8.9 | 7.1 | 52.4 | 41.7 | 71.0 | 4.5 |

**Supplementary Table 5.** Values of the 12 quantitative bromatological traits of leaves of the 21 sorghum studied ecotypes at the maturity stage. *DM* Dry matter (%), *NDF* Neutral detergent fiber (% DM), *ADF* Acid detergent fiber (% DM), *ADL* Acid detergent lignin (% DM), *CF* Crude fiber (% DM), *EE* Ether extract (% DM), Ash (% DM), *CP* Crude protein (% DM), *NFE* Nitrogen-free extract (% DM), *IVEOMD* *in vitro* enzymatic organic matter digestibility (%), *IVTD* *in vitro* true digestibility (%), *ME* metabolizable energy (KJ/kg DM);

| **Ecotype** | **DM** | **NDF** | **ADF** | **ADL** | **CF** | **EE** | **Ash** | **CP** | **NFE** | **IVEOMD** | **IVTD** | **ME** |
| --- | --- | --- | --- | --- | --- | --- | --- | --- | --- | --- | --- | --- |
| E1 | 84.7 | 65.8 | 39.4 | 23.4 | 36.6 | 1.0 | 4.1 | 2.2 | 56.0 | 25.7 | 53.3 | 2.3 |
| E2 | 85.3 | 79.1 | 38.5 | 26.1 | 39.4 | 1.4 | 5.8 | 1.1 | 52.4 | 20.8 | 51.7 | 2.2 |
| E3 | 91.5 | 68.5 | 41.1 | 32.6 | 41.3 | 1.6 | 4.2 | 2.6 | 50.3 | 28.0 | 53.2 | 2.2 |
| E4 | 91.4 | 71.0 | 44.4 | 31.4 | 46.3 | 1.0 | 4.6 | 2.2 | 46.0 | 26.4 | 65.9 | 3.9 |
| E5 | 91.3 | 81.3 | 47.5 | 37.7 | 47.6 | 0.7 | 5.4 | 2.5 | 43.8 | 16.6 | 72.0 | 1.8 |
| E6 | 84.5 | 82.1 | 49.0 | 27.9 | 42.1 | 0.9 | 6.7 | 2.1 | 48.2 | 29.0 | 63.0 | 3.4 |
| E7 | 92.9 | 71.2 | 42.1 | 28.3 | 36.2 | 1.0 | 3.5 | 1.9 | 57.4 | 22.7 | 67.4 | 2.8 |
| E8 | 89.6 | 80.9 | 51.2 | 31.2 | 43.1 | 1.3 | 5.5 | 2.6 | 47.5 | 20.7 | 71.1 | 2.0 |
| E9 | 89.9 | 69.1 | 51.8 | 31.5 | 32.5 | 0.9 | 7.2 | 2.0 | 57.4 | 23.0 | 68.4 | 3.8 |
| E10 | 86.5 | 73.7 | 46.9 | 30.4 | 35.4 | 1.4 | 5.3 | 1.1 | 56.9 | 20.9 | 55.6 | 2.0 |
| E11 | 87.8 | 66.3 | 49.4 | 25.2 | 34.8 | 1.1 | 5.6 | 2.2 | 56.2 | 23.9 | 64.0 | 2.7 |
| E12 | 93.8 | 73.9 | 41.7 | 28.2 | 47.6 | 1.3 | 4.4 | 2.8 | 44.0 | 29.5 | 71.5 | 4.2 |
| E13 | 90.0 | 72.2 | 49.1 | 26.4 | 44.6 | 0.7 | 4.8 | 2.6 | 47.3 | 28.7 | 55.8 | 2.7 |
| E14 | 89.3 | 77.0 | 42.5 | 25.0 | 40.6 | 1.0 | 5.1 | 3.5 | 49.8 | 20.4 | 51.4 | 2.3 |
| E15 | 88.5 | 73.4 | 45.0 | 30.9 | 47.0 | 1.7 | 4.7 | 2.4 | 44.2 | 26.3 | 64.4 | 3.1 |
| E16 | 91.1 | 73.1 | 40.9 | 22.0 | 41.5 | 1.0 | 6.4 | 1.9 | 49.2 | 27.2 | 56.6 | 3.6 |
| E17 | 88.2 | 65.4 | 43.7 | 21.9 | 42.4 | 1.3 | 3.4 | 2.3 | 50.7 | 26.1 | 60.6 | 2.5 |
| E18 | 86.4 | 66.2 | 48.6 | 26.2 | 35.0 | 1.2 | 4.1 | 2.5 | 57.2 | 27.5 | 55.5 | 3.3 |
| E19 | 91.5 | 64.6 | 44.8 | 23.2 | 37.3 | 1.4 | 4.9 | 3.5 | 52.8 | 27.2 | 61.7 | 2.4 |
| E20 | 85.5 | 62.4 | 46.8 | 27.1 | 37.3 | 1.2 | 6.5 | 3.0 | 52.0 | 27.0 | 59.0 | 2.6 |
| E21 | 85.4 | 70.8 | 47.4 | 27.1 | 39.7 | 0.9 | 4.8 | 3.3 | 51.3 | 20.8 | 55.5 | 2.3 |

**Supplementary table 6.** Values of the 12 quantitative bromatological traits of stems of the 21 sorghum studied ecotypes at the maturity stage. *DM* Dry matter (%), *NDF* Neutral detergent fiber (% DM), *ADF* Acid detergent fiber (% DM), *ADL* Acid detergent lignin (% DM), *CF* Crude fiber (% DM), *EE* Ether extract (% DM), Ash (% DM), *CP* Crude protein (% DM), *NFE* Nitrogen-free extract (% DM), *IVEOMD* *in vitro* enzymatic organic matter digestibility (%), *IVTD* *in vitro* true digestibility (%), *ME* metabolizable energy (KJ/kg DM);
